# Protein mediators of chronic kidney disease in Type 2 diabetes: A mendelian randomization study

**DOI:** 10.1371/journal.pmed.1004802

**Published:** 2026-09-11

**Authors:** Kevin Y. H. Liang, Thomas M. Zheng, Dandan Tan, Takayoshi Sasako, Yann Ilboudo, Yiheng Chen, Guillaume Butler-Laporte, Satoshi Yoshiji, J. Brent Richards

**Affiliations:** 1 Lady Davis Institute for Medical Research, Jewish General Hospital, Montréal, Québec, Canada; 2 5 Prime Sciences Inc, Montréal, Québec, Canada; 3 Quantitative Life Sciences, McGill University, Montréal, Québec, Canada; 4 Tanaka Diabetes Clinic Omiya, Omiya, Saitama, Japan; 5 Okachimachi Ohisama Clinic, Tokyo, Japan; 6 Department of Diabetes, Metabolism and Endocrinology, Ichikawa Medical Center, International University of Health and Welfare, Ichikawa, Chiba, Japan; 7 Channing Division of Network Medicine, Department of Medicine, Brigham and Women’s Hospital, Boston, Massachusetts, United States of America; 8 Harvard Medical School, Boston, Massachusetts, United States of America; 9 Precision Healthcare University Research Institute (PHURI), Queen Mary University of London, London, United Kingdom; 10 Centre for Human Genetics, University of Oxford, Oxford, United Kingdom; 11 Division of Infectious Diseases, McGill University Health Centre, Montréal, Québec, Canada; 12 Department of Human Genetics, McGill University, Montréal, Québec, Canada; 13 Programs in Metabolism and Medical & Population Genetics, The Broad Institute of MIT and Harvard, Cambridge, Massachusetts, United States of America; 14 Department of Epidemiology, Biostatistics and Occupational Health, McGill University, Montréal, Québec, Canada; 15 Department of Twin Research, King’s College London, London, United Kingdom; 16 Department of Medicine, McGill University, Montréal, Quebec, Canada; Shanghai Jiao Tong University Affiliated Sixth People’s Hospital, CHINA

## Abstract

**Background:**

Chronic kidney disease (CKD) occurs in 20–50% of the people living with Type 2 diabetes (T2D) and is the leading cause of kidney failure worldwide. The cause of CKD is not fully understood, and few interventions prevent CKD in individuals living with diabetes. Here, we use large-scale proteomics data to identify circulating proteins that mediate the relationship between T2D and kidney disorders.

**Methods and findings:**

First, we used two-sample mendelian randomization (MR) and identified 71 circulating proteins whose levels were altered by genetic predisposition to T2D based on circulating proteomic GWAS from deCODE with 35,559 individuals and T2D GWAS with 80,154 cases. Then, we used *cis*-genetic variants to proxy the causal effect of some of these T2D-influenced circulating proteins and found that, collectively, five proteins (INHBC, GNPTG, LPO, AGRN, and CTSD) affected three kidney traits (blood urea nitrogen [BUN], estimated glomerular filtration rate [eGFR] and CKD risk) based on GWAS with up to 1,004,040 participants. Notably, we found that higher levels of circulating INHBC protein were estimated to lead to a lower eGFR and higher BUN based on MR analyses. We then replicated this MR analysis with proteomic GWAS from four additional cohorts, namely, UKB-PPP, Fenland, ARIC, and EPIC-Norfolk. We observed a consistent direction of effect across all four proteomic GWAS datasets, supporting the robustness of our results against platform and cohort variation. In observational analyses, increased circulating INHBC levels were associated with increased hazard for kidney disease diagnosis in 37,854 UK Biobank participants. We estimated that circulating INHBC levels mediate 1.3% (95% confidence interval [0.85%, 1.9%]) of the association between T2D and kidney disease diagnosis. There are important limitations in this study. Firstly, although we observed limited evidence for violations to the MR assumptions, some are untestable. Secondly, our study was not based on individuals with diabetic kidney diseases, but rather independent population-based studies assessing diabetes and kidney function separately. Therefore, additional functional analyses in disease specific cohort are needed.

**Conclusions:**

Collectively, these findings suggest that T2D influences the risk of CKD, in part, through increased circulating INHBC levels.

## Author summary

**Why was this study done?:**

- Individuals living with diabetes are at an increased risk of developing kidney diseases; however, it is not clear how T2D causes kidney function decline, and more importantly, why this develops in some individuals but not others.
- A better understanding of the pathophysiology of this disease will help develop better therapeutics and improve risk prediction.
- We hypothesize that T2D-mediated changes in the proteome contributes to kidney function decline.

**What did the researchers do and find?:**

- We leverage population-level genetic association studies to identify potential protein mediators between T2D on kidney diseases.
- We identified 5 proteins whose levels were changed by T2D susceptibility that in turn affect kidney function.
- Notably, T2D susceptibility was associated with an increase in circulating inhibin beta C-chain level (INHBC), a member of the TGF-β signaling pathway with known roles in tissue fibrosis.

**What do these findings mean?:**

- We identified 5 proteins (NHBC, GNPTG, LPO, AGRN, and CTSD), whose circulating levels were altered by genetic susceptibility to T2D which in turn affected three kidney traits (BUN, eGFR, and CKD risk).
- Follow-up analyses suggest that T2D may impair kidney function partly through up-• regulating INHBC levels.
- However, there are important limitations to this study. Namely, this study infers protein mediators between T2D and kidney disease using disease-specific GWAS for T2D and kidney function measurements and not diabetic kidney disease specifically; therefore, follow-up studies on a diabetic kidney disease-specific cohort are needed for validation of the findings in this study.

## Abstract 3

Kevin Liang and colleagues used large-scale genetic and proteomic data to explore how Type 2 diabetes may be linked to chronic kidney disease, identifying several circulating proteins associated with kidney health. Their analyses highlighted INHBC as a notable candidate.

## 1 Introduction

Chronic kidney disease (CKD) occurs in 20–50% of diabetic patients and is the leading cause of kidney failure worldwide [1–3]. Kidney failure is marked by the need for renal replacement therapy (e.g., dialysis or kidney transplant) for survival. Importantly, treatment options are limited once patients progress to kidney failure. Kidney transplants are potential treatments for diabetic patients with kidney failure; however, these procedures come with significant risks. Thus, early intervention to significantly slow down or halt the progression of kidney function decline and ultimately reduce the need for kidney transplant is crucial.

CKD in people with diabetes is caused by a diverse network of pathophysiological mechanisms. Studies have suggested various cytokines, hemodynamics changes (e.g., renin-angiotensin-aldosterone activation), inflammation, fibrosis, oxidative stress, podocyte injury and hyperglycemia to play a role in this disease [4]. However, it is not clear why some individuals with diabetes develop kidney disease while others do not. Currently, five options are available for slowing down the progression of CKD in people with diabetes: Renin-angiotensin system (RAS) inhibitors [5], drugs acting as mineralocorticoid receptor antagonists [6], *SGLT2* inhibitors [7,8], and *GLP1R*-agonists [9], in addition to control of relevant risk factors (e.g., blood pressure and glucose control) [10]. Despite these interventions, some people with diabetes continue to progress to kidney failure [8,9,11]. This underscores the need for more effective treatment options and, therefore, a better understanding of disease pathophysiology.

Diabetes is a complex disease and likely has a broad impact on the proteome. Proteins are therefore potential mediating factors in the development or progression of CKD in people with diabetes. Moreover, identifying disease-causal factors could help in the development of novel drugs, the repositioning of existing drugs, or the identification novel biomarkers to predict disease progression. Consequently, one way to understand the causes of CKD is to study the role of circulating proteins. Recent releases of population-wide proteogenomic studies have provided the foundation to assess the causal role of circulating proteins in the development of diseases such as Coronavirus disease 2019 (COVID-19) and cardiometabolic diseases, to name a few [12,13]. These proteome-wide studies have identified potential drug targets and provided insights into possible interventions.

By applying proteogenomics through the lens of mendelian randomization (MR), we can estimate if circulating proteins play a causal role in disease susceptibility, which sometimes can help identify therapeutic targets. MR is an established genetic epidemiology technique that assesses causal relations by using genetic variants as a proxy for exposure to risk factors [14]. It often relies on well-powered genome-wide association studies (GWAS) to identify genetic variants that are reliably associated with the exposure. Importantly, because genetic variants are assigned randomly at conception and always precede disease onset, causality can be inferred if at least three key assumptions are met. First, the genetic variants must be strongly associated with the exposure of interest. Second, the genetic variants must not be associated with the confounders of the exposure and outcome of interest (i.e., confounders of the relationship between type 2 diabetes (T2D) and circulating protein levels, and between circulating protein levels and kidney disease). Last, the genetic variants must influence the outcome only through the exposure of interest. Of these, the last, also known as directional horizontal pleiotropy, is the most problematic assumption and often leads to spurious results in MR. However, in the case where the exposure is a circulating protein, we can use genetic variants that are near the gene (i.e., *cis-*protein quantitative loci [*cis-*pQTL]). The proximity of the *cis-*pQTLs to the gene of interest reduces the chance that the *cis-*pQTLs have strong effects on the outcome independent of their effects on the circulating protein levels. Hence, proteins with *cis-*pQTLs are especially well-suited for MR studies, where the circulating protein levels are the exposure of interest.

**Fig 1:**
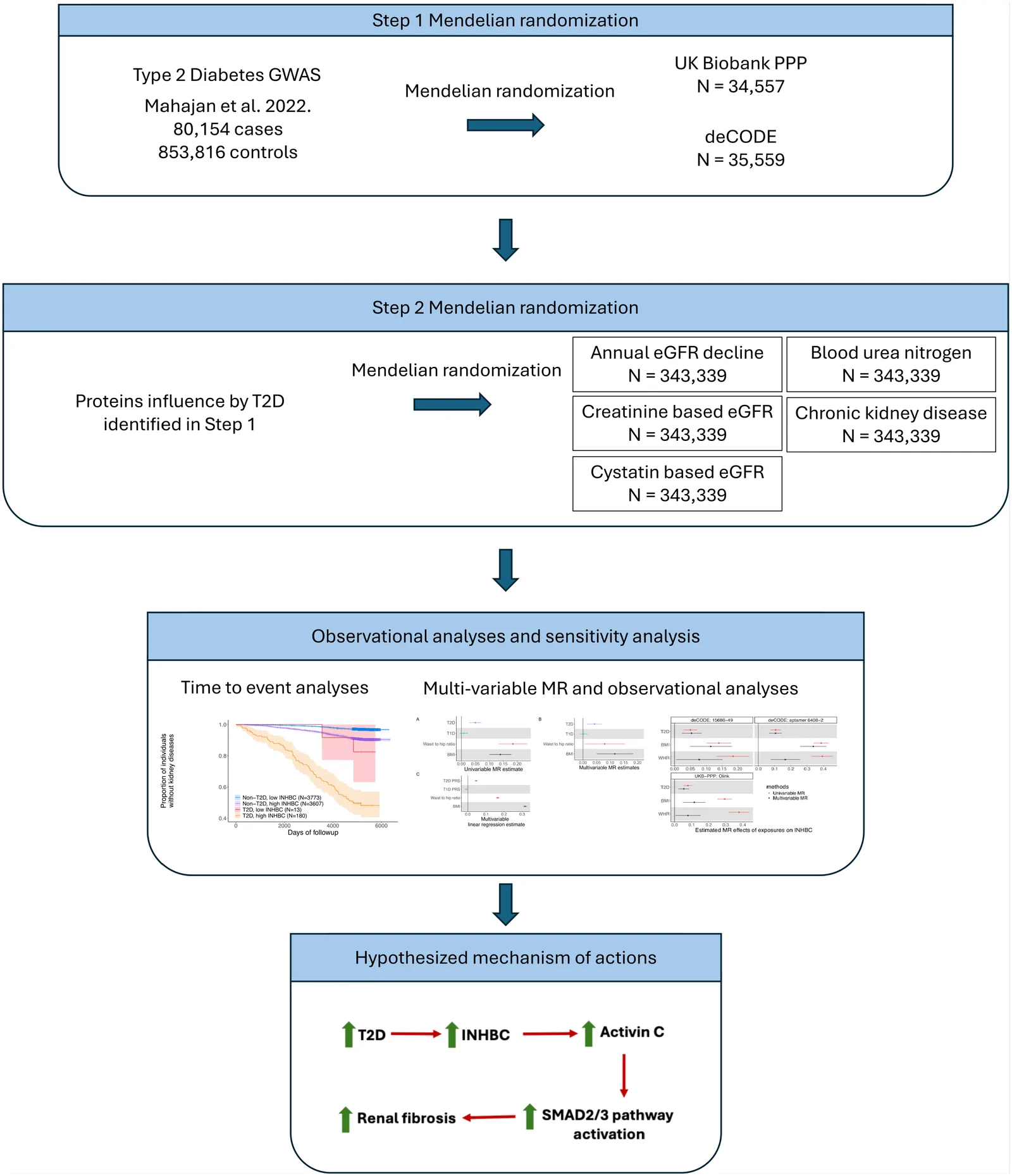
Study overview.

In this study, we used two-sample MR in large population-based cohorts to identify circulating proteins influenced by genetic susceptibility to T2D and which of these proteins, in turn, influence the risk of kidney disease (Fig 1). We first aimed to identify proteins whose levels were influenced by genetic susceptibility to T2D. We then assessed the effects of these proteins on kidney diseases and related traits to determine which proteins are likely to impair kidney function.

## 2 Methods

### 2.1 Study population

MR relies on well-powered GWAS. GWAS sources for the traits used in this study are outlined in S1 Table. Proteomics GWAS summary statistics were obtained from five publicly available sources, all of which were based primarily on individuals of European ancestry, which provides the largest sample sizes and reduces potential bias due to population stratification. The five sources of proteomics GWAS were: deCODE (maximum sample size: 35,559), UK Biobank Pharma Proteomics Project (UKB-PPP) (maximum sample size: 34,557), Fenland (maximum sample size: 10,708), European Prospective Investigation of Cancer (EPIC)-Norfolk (maximum sample size = 2,887) and Atherosclerosis Risk in Communities (ARIC) (maximum sample size: 7,213) [15–19]. Individual-level data of those in the UKB-PPP were also used for follow-up analyses.

### 2.2 Phenotype definitions among UK Biobank participants

#### 2.2.1 Disease definition.

Kidney and liver disease within the UK Biobank were defined based on combinations of the following: date of first International Classification of Diseases 10th revision (ICD-10) codes, cause of death, date of first Office of Population Censuses and Surveys Classification of Interventions and Procedures version 4 (OPCS-4) codes, and relevant laboratory results as described below. We did not consider self-reports when defining disease status.

Kidney diseases within the UK Biobank were defined based on 18 ICD-10 (UK Biobank field: 41280), and 20 OPCS-4 codes (UK Biobank field: 41282) (S2 Table). Relevant ICD-10 and OPCS-4 codes were collected based on UK Biobank field descriptions to include several kidney complications relevant to diabetes. Individuals with a record of any of the listed ICD-10/OPCS-4 codes in S2 Table were considered to have kidney disease. Incidence and prevalence status were determined by comparing the earliest diagnosis date with the blood draw date (UK Biobank field: 3166) for each participant. Prevalent cases were those whose earliest diagnosis date was before the blood draw date. Incident cases were those whose earliest diagnosis date was after the blood draw date. Individuals may have more than one diagnosis but only diagnoses with known diagnosis dates were considered. Individuals with relevant diagnoses but without any diagnosis dates were dropped from further analyses as prevalent/incident status could not be determined.

Prevalent CKD cases were also defined based on creatinine-based estimated glomerular filtration rate (eGFR_creatinine−_) and urinary albumin-to-creatinine (uACR) ratio. Baseline blood creatinine level, urine creatinine, urine albumin, age at recruitment, and genetically determined sex were obtained from UKB fields 30700, 30510, 30500, 21022, and 22001, respectively. Those with eGFR_creatinine_ less than 60 ml min^−1^ per 1.73 m^2^ or uACR ≥ 3 mg/mmol were considered to have prevalent kidney diseases and were removed from further analyses.

Individuals whose documented causes of death included the same ICD-10 codes used to define kidney disease as outlined in S2 Table were also considered as incident cases. For these samples, the date of death was used as the date of diagnosis if the date diagnosis was not available.

T2D and type 1 diabetes (T1D) diagnoses were defined based on ICD-10 codes E11 and E10, respectively (S2 Table). Like before, prevalence and incidence status were determined by comparing the earliest diagnosis date with the blood draw date for each participant. Prevalent and incident cases were patients whose earliest diagnosis date was before or after the blood draw date, respectively. Individuals with diabetes but without diagnosis date, or those who were diagnosed with both T2D and T1D were removed from subsequent analyses. Individuals without a T2D or T1D diagnosis (based on ICD-10 code), but whose HbA1c level was above 48 mmol/mol (or 6.5%) at baseline were also removed.

Liver diseases were defined as ICD-10 codes K70 to K77. In addition, individuals whose documented cause of death included the same ICD-10 codes used to define liver diseases were considered incident cases.

#### 2.2.2 Anthropometric traits and biomarkers.

eGFR_creatinine_ for all UK Biobank participants was computed using the eGFR equation without the race variable that was published by the Chronic Kidney Disease Epidemiology Collaboration (CKD-EPI) in 2021 [20]. eGFR_creatinine_ was computed using the baseline blood creatinine levels for all UK Biobank participants without missing data (UK Biobank field: 30700). The urinary albumin-to-creatinine ratio was computed using baseline measurements (UK Biobank fields: 30510, 30500, respectively).

Waist-to-hip ratio (WHR) was calculated from waist and hip circumference data (UK Biobank fields: 48 and 49, respectively).

### 2.3 Reference panel

Imputed data from a random subset of 5,000 individuals of European ancestry were used as the reference panel for all analyses in this study. Subsets were generated as required and consequently each analysis may use different subsets of the UK Biobank.

### 2.4 Proteomics data sources

In this study, we obtained proteomic GWAS from five sources covering the two commonly used proteomic platforms, namely Olink and SomaLogic (S1 Table). Both platforms aim to quantify circulating protein levels but achieve this goal through different means. Consequently, these platforms are subjected to different biases as previously illustrated by Eldjarn and colleagues [21]. By incorporating convergent proteomic GWAS based on different technologies, we can be more confident that our results reflect true biology and are robust to platform and cohort-specific factors.

Protein quantitative trait loci (pQTL) from deCODE were obtained from the original 2021 publication for the data release [15]. Only conditionally independent *cis-*pQTLs as defined by the authors of the original deCODE data release were kept. Details regarding measurements and preparation of protein levels for deCODE GWAS are provided by Ferkingstad and colleagues in the original data release [15]. Briefly, it was measured using the SomaScan v4 aptamer binding assay (SomaLogic, Boulder, CO). The SomaScan platform is an aptamer-based assay where each aptamer targets a specific region of a protein. Multiple aptamers may target the same protein. For clarity, we will refer to the quantification of SomaScan aptamers as the levels of proteins unless otherwise specified. We further restricted to single nucleotide polymorphisms (SNPs) only pQTLs that we could lift over to build GRCh37. In total, we have *cis-*pQTL for 1,721 proteins. A full list of proteins and the corresponding aptamers evaluated in this study is listed in S3 Table.

We obtained phase 2 release of the UKB-PPP GWAS summary statistics for 2,923 proteins measured in 34,557 individuals from the original publication [16]. Protein level quantification and quality control are described previously [16]. Briefly, protein levels were quantified by the anti-body-based Olink assay (Olink, Uppsala, Sweden). The proteins were measured across eight protein panels. The values were then normalized and corrected for batch and plate effects. Cis-pQTLs for the Olink proteomics GWAS were obtained from the original publication [16], which were identified using sum of single-effects regression (SuSiE) [22]. SuSiE is a statistical fine-mapping tool aimed at identifying potential causal variants within a region. Sun and colleagues also confirmed statistical independence using multivariable linear regression [16].

We also obtained proteomics GWAS summary statistics from the Fenland, ARIC, and EPIC-Norfolk cohorts [17–19]. The European ancestry summary statistics were used for the ARIC cohort. The Fenland and ARIC cohorts used the SomaScan aptamer binding assay to measure protein levels. Cis-pQTLs for the Fenland and ARIC cohorts were defined in this study as independent genome-wide significant variants within ±500 kilobase pairs region around the transcript as stated on the UCSC genome browser (INHBC, GRCh37: chr 12, 57,828,543–57,844,609). Independent genome-wide significant variants were identified by clumping using Plink v1.9.0 (see Methods: Exposure GWAS pre-processing for further detail). The EPIC-Norfolk cohort used the Olink anti-body-based platform to measure protein levels. Independent *cis-*pQTLs for INHBC were defined by the authors in the original publication. In all cases, only *cis-*pQTLs were used in MR analyses.

We did not perform MR to assess the effect of T2D on INHBC levels in the ARIC cohort because the released summary statistics contained only the SNPs in the *cis-*region of INHBC. As a result, there was no variant overlap between the T2D instruments and the INHBC GWAS summary statistics from ARIC, making such analyses impossible.

### 2.5 Mendelian randomization

We used two-sample MR in this study for two purposes. Firstly, MR was used to identify circulating proteins whose levels were affected by genetic susceptibility to T2D. Secondly, we used MR to determine whether any of the proteins whose levels were altered by T2D in turn affected kidney traits. Two-sample MR makes use of GWAS summary statistics from two different cohorts, which allowed us to take advantage of GWAS with the largest sample sizes for each trait thus providing more statistical power to detect causal relationships, should one exist. For relevant kidney traits, we selected the largest GWAS of predominantly European ancestry that were publicly available at the start of the study (S1 Table).

There are important limitations to MR that should be considered when interpreting the results. Firstly, by using genetic variants to proxy exposure levels, MR assumes lifelong exposure to the risk factor; therefore, the estimated causal effects would represent the effect of exposure over one’s lifetime. Secondly, MR relies on three key assumptions, as outlined above, some of which can only be assessed via sensitivity analyses and cannot be ruled out completely. Thirdly, two-sample MR can be performed based on any traits with GWAS summary statistics; however, basic criteria to establish causality are required. For example, the populations in the outcome and exposure cohorts are assumed to be exposed to the same exposure at a similar level, and the effects of the genetic determinants are assumed to be the same. The latter assumption is often satisfied by restricting the analyses to individuals of the same genetic ancestry, hence similar genetic backgrounds.

#### 2.5.1 Outcome GWAS pre-processing.

GWAS of individuals of predominantly European ancestry were obtained from public sources, which provided the largest sample sizes and reduced potential bias due to population stratification. All outcome GWAS were processed as follows. SNP positions were converted to GRCh37 with the standalone LiftOver tool when needed (https://www.genome.ucsc.edu/cgi-bin/hgLiftOver). Effective sample sizes for case/control studies were computed as $N_{effective}=\frac{\text{2}}{\left(\frac{\text{1}}{case}+\frac{\text{1}}{control}\right)}$ as previously described [23]. Non-ACTG variants were removed. After pre-processing, all GWAS formatted by the *format_data()* function from the TwoSampleMR R package v0.5.7.

#### 2.5.2 Outcome GWAS selection and description.

We selected four traits related to CKDs to assess the function of T2D on kidney functions (S1 Table). These traits were selected because they are well-established markers for kidney function. Details for each GWAS is provided in the original publication. In particular, we note that eGFR declined was computed as $\frac{-\text{1}*(eGFR_{followup}-eGFR_{baseline})}{Number\;of\;years\;of\;follow\;up}$, thus a positive number corresponds to the magnitude of decline [24]. eGFR decline GWAS was adjusted for age, sex, and diabetes mellitus status, although the author noted that these can be seen as equivalent to adjusted for age and sex only (https://www.uni-regensburg.de/medizin/epidemiologie-praeventivmedizin/genetische-epidemiologie/gwas-summary-statistics/index.html) [24]. CKD status in the CKD GWAS was defined as individuals with eGFR level less than 60 ml min^−1^ per 1.73 m^2^ [25].

#### 2.5.3 Exposure GWAS pre-processing.

Genetic instruments for deCODE, UKB, and EPIC-Norfolk protein GWAS were identified in the original publications [15–17]. Genomic positions for all genetic instruments were lifted over to GRCh37 with the standalone LiftOver tool when needed (https://www.genome.ucsc.edu/cgi-bin/hgLiftOver) and non-ACTG variants were removed. Genetic instruments for GWAS where the authors did not report *cis-*instruments were identified by clumping using PLINK v1.9 with the following plink parameters: clump-p1 of 5 x 10^−8^, clump-r2 of 0.001, clump-kb of 10,000, maf of 0.001, and geno of 0.1. Due to the complex linkage disequilibrium (LD) structure in the MHC region (GRCh37: chromosome 6, position 28,477,797–33,448,354), it was removed from all MR analyses. Only autosomal variants were kept (i.e., sex chromosomes were not included). Exposure GWAS were formatted with the *format_data()* function from the TwoSampleMR R package v0.5.7.

The transformation for each GWAS is described in the original publications listed in S1 Table. Briefly, the units for CKD, T1D, and T2D are in log odds ratio. Protein levels from all five cohorts, body mass index (BMI), and WHR were inverse rank normal transformed. As such, one unit corresponds to one standard deviation (SD) in relative fluorescence units for proteins measured by the SomaLogic platform and normalized protein expression for the Olink platform. One unit for BMI corresponds to one SD in kg/m^2^ for BMI. eGFR_creatinine_, cystatin-based estimated glomerular filtration rate (eGFR_cystatin_), and blood urea nitrogen (BUN) have been natural log transformed and are in units of ln(ml/min/1.73 m^2^), ln(ml/min/1.73 m^2^), and ln(mg/dL), respectively. eGFR decline was defined as the magnitude of decline per year in units of ml/min/1.73 m^2^/year. Genetic instruments for all exposures used in this study are provided in S4 and S5 Tables.

#### 2.5.4 Mendelian randomization analysis.

Matching of effect sizes and alleles between exposure and outcome GWAS was done with the *harmonise_data()* function of the TwoSampleMR R package with default parameters and modifications to allow for proxy search using local reference panel. Ambiguous alleles (i.e., A/T, C/G) with minor allele frequencies of 0.42 or greater were removed as described (https://mrcieu.github.io/TwoSampleMR/articles/harmonise.html). Otherwise, effect alleles of ambiguous pairs were inferred from effect allele frequencies.

Proxy search was done using the *get_ld_proxies()* function with custom modification of the gwasVCF R package [26] with a window size of 1,000 kilobase pairs around the lead variant, an R^2^ threshold of 0.8. The proxy SNP with the largest R^2^ was selected. In cases where multiple SNPs have the same best R^2^ to the query variant, the one with the shortest distance was selected.

When two or more instruments were available, the random effect inverse variance weighted causal estimates were used as the primary causal effect estimate. If only 1 instrument was available, the Wald ratio was used. MR analyses followed the Strengthening the Reporting of Observational Studies in Epidemiology using mendelian Randomization (STROBE-MR) guideline (S1 Checklist).

### 2.6 Mendelian randomization sensitivity analyses

#### 2.6.1 Assessing weak instrument bias.

Two steps were used to ensure the genetic instruments were strongly associated with the exposures. The first was to select only independent genome-wide significant variants using the standard p-value threshold of 5 x 10^−8^. For the protein GWAS obtained from UKB-PPP, deCODE, or the EPIC-Norfolk cohort, independent *cis-*pQTLs were defined by the authors [15–17]. For all other GWAS, clumping was used to identify independent genome-wide significant variants as described above.

The second method we used to assess weak instrument bias was the cumulative F-statistics. The cumulative F-statistic was computed as $F=\;\frac{R^{\text{2}}*(N-\text{1}-K)}{\left(\text{1}-R^{\text{2}}\right)*K}$, where R^2^ is the cumulative proportion of variance explained, N is the total sample size (or effective sample size for case/control studies) and K is the number of variants [27,28]. The proportion of variance explained by one variant was computed as $R^{\text{2}}=\;\frac{\left(\text{2}*\beta^{\text{2}}*maf*(\text{1}-maf)\right)}{\left(\text{2}*\beta^{\text{2}}*maf*\left(\text{1}-maf\right)\right)+(se_{beta}^{\text{2}}*\text{2}*n*maf*\left(\text{1}-maf\right))}$ and this was summed across all instruments. Instruments without allele frequency information were dropped from the R^2^ calculation.

It is also worthwhile to note that weak instruments tend to bias the results towards the null in two sample MR; therefore, weak instruments are more likely to lead to reduced statistical power rather than false positive results. Although sample overlaps between exposures and outcomes may lead to bias, we have also quantified the maximum relative bias due to such overlap (see Methods: Assess bias due to sample overlap section below).

#### 2.6.2 Assessing horizontal pleiotropic effects.

Unlike the absence of weak instrument bias, the absence of horizontal pleiotropy cannot be completely assessed as it requires knowing all pleiotropic effects of each exposure, which is not known at present. However, some steps can be taken to reduce the probability of directional horizontal pleiotropy such as the use of other causal effect estimates that can tolerate variable extent of horizontal pleiotropic effects, although often with reduced statistical power.

To reduce the chance of horizontal pleiotropy, we first checked whether the *cis-*pQTLs used as instrumental variables were shared by two or more proteins. Although using only *cis-*pQTLs that are unique for each protein will reduce statistical power in detecting causal relations, such instruments are less likely to be affected by horizontal pleiotropy. Additionally, we performed pair-wise conditional colocalization (PWCoCo v3) [29] to assess the probability that the exposure and outcome share the same causal variants within a pre-specified region. For colocalization analysis, *p*-values were computed from effect size and standard error where the square of the Z-score is assumed to follow a chi-squared distribution with one degree of freedom. We performed colocalization for each *cis-*genetic instrument.

We also performed MR-Egger, weighted median, weighted mode, and simple mode estimates for exposures with sufficient instruments. These methods have different tolerance of horizontal pleiotropic effects. For instance, weighted median and weighted mode simply require that the majority of the weighted effects or the largest subset of weighted effects originate from valid instruments, respectively. This is a more relaxed assumption than the inverse variance weighted estimate which assumes all effects originate from valid instruments. Consistent direction of effects across different methods will provide greater confidence that the relationship observed between exposure and outcome was not due to horizontal pleiotropy.

When potential horizontal pleiotropic effects are known, it is also possible to partially account for these effects in a multivariable MR (MVMR) setting. In MVMR, the direct causal effects of exposures on outcome can be assessed provided that three assumptions are satisfied: the selected instruments are related to one or more of the risk factors, the instruments are not associated with the confounders of the outcome and risk factors, and the instruments only influence the outcome through the risk factors [30]. Exposure and outcome GWAS were formatted and harmonized with the TwoSampleMR R package v0.5.7. Instrument selection was done using the TwoSampleMR *mv_extract_exposures_local()* function with modifications to perform clumping using a local reference panel with a p-value threshold equivalent to a multiple testing corrected threshold for all exposures (i.e., 5 x 10^−8^/number of exposure). We also enforced a stricter clumping threshold of r^2^ = 0.0001. Although these criteria will reduce the number of instruments selected, we are more likely to retain the strongest independent instruments across exposures and minimize the risk of weak instrument bias which is more likely in the MVMR setting working with pleiotropic traits such as BMI and WHR. To assess heterogeneous effects and instrument strength in an MVMR setting, a phenotype correlation matrix was required to estimate genetic correlation due to sample overlap. We computed this matrix using the *estimateSyy()* function implemented in metaCCA R package v1.22.0 [31], which allowed us to estimate phenotype correlations using only GWAS summary statistics [12,32]. We used all variants with a minor allele frequency of 1% or more regardless of significance as recommended by the authors [31]. The MHC region was removed for both instrument selection and the estimation of phenotype correlation to avoid bias driven by large LD blocks within this region.

#### 2.6.3 Assess reverse causation and heterogeneity.

We used two methods to assess reverse causation: bi-directional MR and the Steiger test. Only MR results that were significant in one direction, after multiple testing correction were kept. Bonferroni correction was done for each direction in each step separately. A Bonferroni corrected *p*-value threshold of 0.05 was used to determine statistical significance. For step 1, we performed 1,721 forward MR (i.e., T2D on proteins) and 1,517 reverse MR (i.e., proteins on T2D) to identify proteins that were likely in the right causal direction (i.e., T2D influences protein levels). The smaller number of reverse MR analyses was due to the lack of *cis-*pQTLs and proxies for some proteins in the T2D GWAS. For step 2, we performed 278 forward MR (i.e., proteins on kidney function) and 284 reverse MR (i.e., kidney function on proteins) to assess the causal relations between 4 kidney phenotypes (eGFR, BUN, eGFR decline, CKD) and the 71 T2D altered proteins identified in step 1.

We also performed the Steiger test for selected MR results to assess reverse causation. The Steiger test assesses whether the variance of the exposure explained by the genetic variants is greater than the variance of the outcome explained by the same genetic variants. This can be expected if the causal direction (i.e., genetic variant affects the outcome through the exposure) is true.

To test for heterogeneous effects, we used the *mr_heterogeneity()* or *pleiotropy_mvmr()* function from the TwoSample MR v0.5.7 or MVMR v0.4 packages for univariable and multivariable MR, respectively. The I^2^ statistics were calculated as $I^{\text{2}}=\;\frac{\left(Q-Q_{df}\right)}{Q}*\text{1}\text{0}\text{0}$ in both cases. MR results with an I^2^ of less than 50, or where the Q-statistic *p*-value was > 0.05 were assumed to show low heterogeneous causal effects among instrumental variables. This was done for exposures with two or more instruments.

#### 2.6.4 Assess bias due to sample overlap.

Sample overlap between exposures and outcome GWAS, together with weak instruments may introduce bias in the MR estimates. The maximum relative bias can be estimated by $relative\;bias=\;\phi \frac{\text{1}}{F}$, where ϕ represents the proportion of overlaps and F is the cumulative F-statistics [12,33]. We computed the maximum relative bias by assuming maximum sample overlap and minimum F-statistics among the relevant MR findings. The relative bias can be interpreted as the bias observed in the MR estimate relative to the bias observed in the observational estimate.

#### 2.6.5 MR replication.

Circulating INHBC level is one of the biomarkers identified to be a potential mediator for the effect of T2D on kidney function in this study. Based on existing literature and the possible role of INHBC in kidney function [34–36], we decided to conduct several follow-up and sensitivity analyses focusing on this protein.

We replicated the MR analyses of selected exposures and outcomes in four independent proteomics cohorts: deCODE, Fenland, EPIC-Norfolk, and ARIC. *cis-*pQTLs for deCODE, UKB-PPP, and EPIC-Norfolk were selected by the authors in the original publication [15,16,18]. Instrumental variables for the Fenland and ARIC cohorts were selected by clumping and then filtered to include only variants within 1 megabase pair of the transcript region as described above. We additionally removed *cis-*pQTLs with a minor allele frequency of less than 1%. Overall, one instrument was selected for both INHBC in the deCODE and EPIC-Norfolk cohorts. Three instruments were selected in the ARIC cohort. Two instruments were selected for the UKB cohort. Eight instruments were selected in the Fenland cohort (S6 Table).

#### 2.6.6 Colocalization analysis with GTEx v8 eQTL.

We performed colocalization analysis using PwCoCo to assess whether the INHBC pQTL instrument is shared with inhibin beta E-chain protein (INHBE) or R3H domain-containing protein 2 (R3HDM2) eQTLs in two tissues: liver and whole blood. Liver tissue was selected for this analysis as it is the primary site for INHBC production, and we selected whole blood as the deCODE pQTL data is based on circulating levels of INHBC. We used European specific eQTL data from v8 of GTEx to be consistent with deCODE GWAS. Sample size of the eQTL data was computed based minor allele counts and minor allele frequencies. As effect allele frequencies were not reported in GTEx v8, we restricted the analysis to only nonpalindromic variants and used the allele frequencies obtained from deCODE pQTL. Given that both GWAS datasets are based on individuals of European ancestry, allele frequencies are expected to be comparable. Regional locus plots were created using the locuszoomr R package [37].

### 2.7 Observational analyses

#### 2.7.1 Time-to-event analysis.

Kaplan-Meier (KM) curves were fitted using the ggsurvfit v3.6.4 R package with default parameters. KM curves are a way to visualize the relative risk of developing diseases between different groups of individuals while accounting for the time of diagnosis. It is a plot of the proportion of individuals without kidney disease diagnoses as a function of days of follow-up. Endpoints were defined as one of the following, whichever comes first: date of earliest incident kidney diagnosis, date of death, or the last update of ICD-10 records (September 2023). Date of death data were obtained from UKB fields 40,000. We compared four groups of individuals: individuals living with T2D with low circulating INHBC levels, individuals living with T2D with high circulating INHBC levels, individuals without T2D with low circulating INHBC levels, and individuals without T2D with high circulating INHBC levels. High and low protein levels were defined as the top and lower 10th percentile respectively. We further restricted the analyses to individuals without T2D or those with T2D diagnoses before blood draws (i.e., only prevalent T2D cases or those without T2D diagnoses and no incident T2D cases were included) to evaluate the association of kidney complications because of T2D. We also removed individuals with any liver diseases (prevalent or incident cases) to reduce the potential impact of liver function on INHBC levels as INHBC is predominantly produced by the liver.

One limitation of the KM curve is that it is difficult to control for the effects of other factors, such as age, on the risk of developing the disease. Therefore, we fitted a multivariable Cox proportional hazard model for the risk of developing kidney disease with BMI, WHR, INHBC levels, T2D status, age, genetically predicted sex (i.e., sex predicted based on genetic data rather than self-report), and interactions between T2D and INHBC levels to determine the effects of each of these factors. We also included genetically predicted ancestry to account for confounding due to ancestry, which was obtained from a prior publication [38]. One important assumption of this model is that it assumes a constant hazard ratio regardless of exposure time (e.g., the difference in risk of developing the disease between males and females is constant across time).

#### 2.7.2 Ethics statement.

This research program using anonymized individual level UK Biobank data was conducted under IRB number 2024-38466 approved by the research ethics board of CIUSSS West-Central Montreal Board. This research has been conducted using the UK Biobank Resource under Application Number 27449 starting in 2024.

#### 2.7.3 Large language model use.

ChatGPT (OpenAI) and Grammarly (Grammarly Inc) were used to check grammar and improve writing style for clarity and conciseness. ChatGPT (OpenAI) and Google Gemini were used for troubleshooting analysis codes. The authors review the content of the manuscript and retain responsibility for the data and text presented in this study.

## 3 Results

### 3.1 Step 1 MR: Susceptibility to T2D was associated with changes in the levels of 71 proteins

We used two-sample MR to estimate the causal effect of susceptibility to T2D on 1,721 proteins obtained from the deCODE study (maximum sample size was 35,559) (S1 Table). The protein GWAS were based on inverse normal rank transformed levels as described by Ferkingstad and colleagues [15]. The T2D GWAS was obtained from a large-scale meta-analysis released by the DIAGRAM consortium (*N* cases = 80,154 *N* controls = 853,816, S1 Table) [39].

We used 203 genetic instruments selected for susceptibility to T2D after clumping to account for LD (S4 Table) (see Methods for further detail). The cumulative F-statistics was 80, which indicated a limited risk of weak instrument bias (S3 Table).

**Fig 2:**
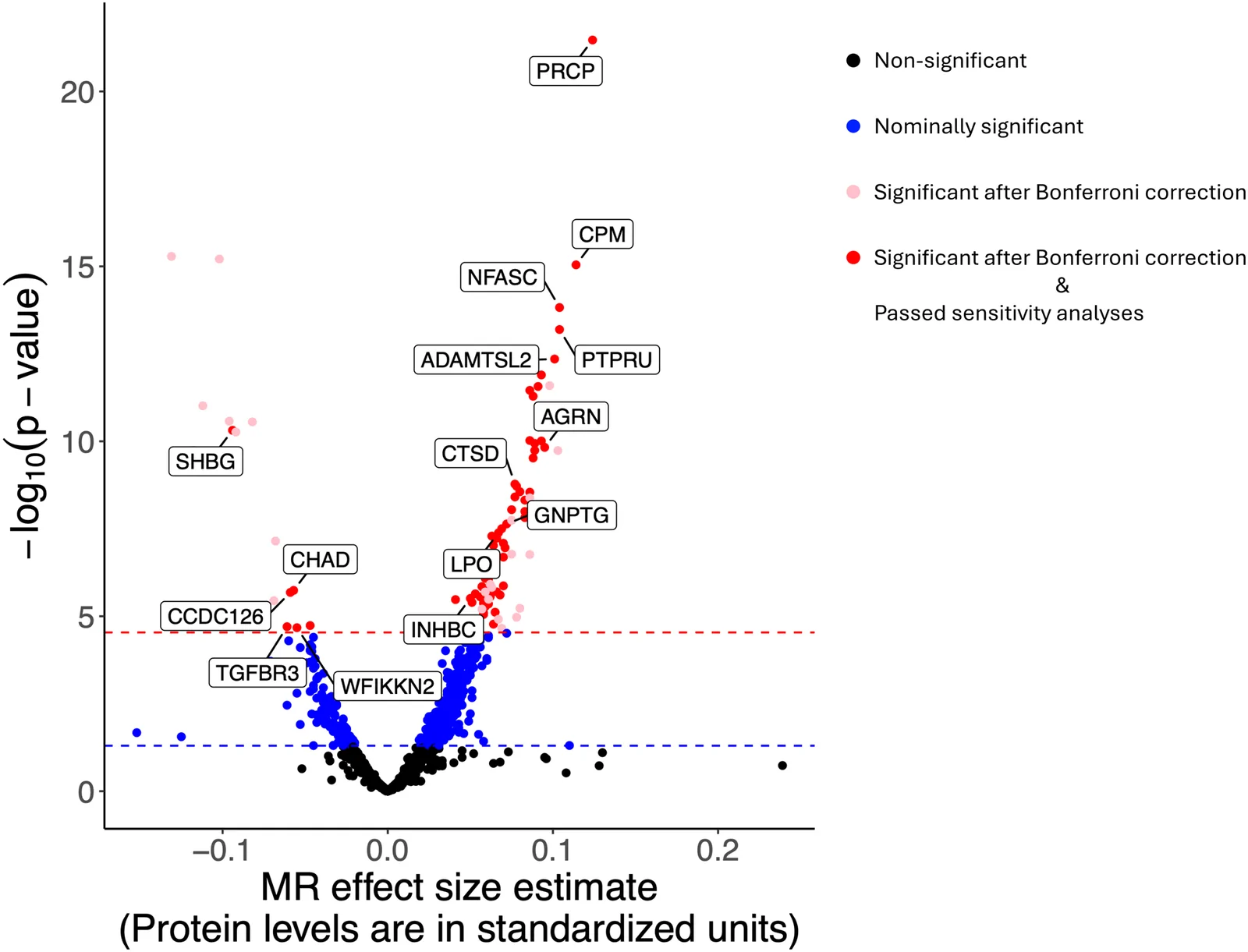
Summary of two-sample mendelian randomization results. Step 1: two-sample MR results of the estimated MR effects of susceptibility to T2D on the 1,721 deCODE proteins. Black dots are nonsignificant results. Blue dots are nominally significant (unadjusted *p*-value < 0.05). Red and pink dots are significant results after Bonferroni correction. Red dots are significant results after Bonferroni correction that also passed sensitivity analyses. Protein levels were inverse rank normal transformed. Units for T2D are in log odds ratio. The top 10 proteins that were most strongly affected by T2D and the 5 proteins that influenced kidney functions are labeled. Protein names are listed in S3 Table.

Of the 1,721 proteins tested, circulating levels of 71 proteins were significantly altered by susceptibility to T2D after a conservative Bonferroni correction (*p*-value threshold = 0.05/1,721  = 2.91 x 10^−5^) and sensitivity analyses (i.e., reverse causation, test of heterogeneity, F-statistics, and horizontal pleiotropy, see Methods and S1 Text for further details) (Figs 2, S3, S7 Tables). The estimated MR effects for genetic susceptibility to T2D on the 71 proteins ranged from −0.094 SD for Sex hormone-binding globulin (SHBG) to 0.124 SD for Lysosomal Pro-X carboxypeptidase (PRCP) per one log odds increased in T2D susceptibility (S3 Table). The largest absolute MR-Egger intercept value among the 71 proteins was 0.004 (*p*-value = 0.065). This provides evidence for a limited risk of directional horizontal pleiotropy, given that an intercept value of zero would indicate balanced horizontal pleiotropy or lack of horizontal pleiotropy.

### 3.2 Step 2 MR: Circulating level for 5 of the 71 T2D-altered proteins influenced 3 kidney traits

We then tested whether any of the 71 circulating proteins whose levels were influenced by genetic susceptibility to T2D, in turn, influence any of the 4 kidney-related phenotypes (CKD, eGFR_creatinine_, annual eGFR decline, and BUN). GWAS for these traits were obtained from publicly available GWAS of predominantly European ancestry individuals (S1 Table). The protein GWAS were obtained from the deCODE study; the same as what was used in Step 1. The median F-statistic across all MR analyses in this step was 341 (min: 34, max: 1,614) which again suggested a limited risk of weak instrument bias (S8 Table).

**Fig 3:**
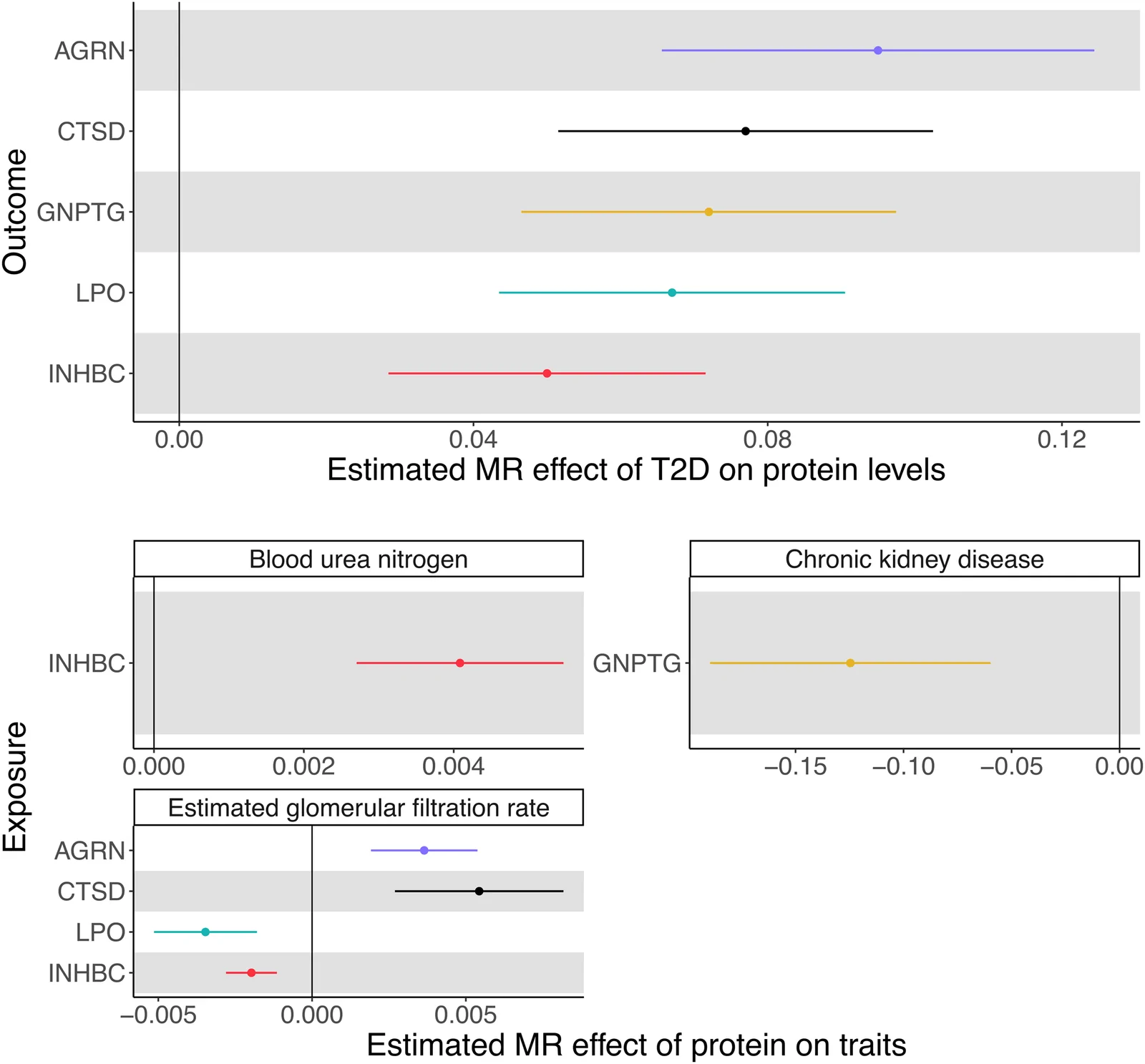
MR result summaries of proteins estimated to be causally influenced by T2D which, in turn, have significant MR effects on three kidney traits. The unadjusted *p*-values and 95% confidence intervals are shown. Vertical lines indicate null effects. Protein levels were inverse normal rank transformed. Units for blood urea nitrogen, estimated glomerular filtration rate, and chronic kidney disease are ln(mg/dL), ln(ml/min/1.73 m^2^), and log odds ratio respectively. ln: natural log transformation. INHBC: Inhibin beta C-chain, AGRN: Agrin, CTSD: Cathepsin D, LPO: Lactoperoxidase.

278 protein-to-disease MR assessments were performed to assess the effects of the 71 proteins identified previously on 4 kidney outcomes. Certain protein-disease pairs could not be assessed due to the lack of genetic instruments. Six of the 278 MR results were significant after a conservative Bonferroni correction (p-value threshold: 0.05/278 = 1.8 x 10^−4^) and sensitivity analyses (S7, S8 Tables; see Methods and S1 Text for further details). This included the effects of four proteins (Agrin [AGRN], Cathepsin D [CTSD], Lactoperoxidase [LPO], and INHBC) on two kidney function biomarkers (eGFR_creatinine_ and BUN) and one protein (N-acetylglucosamine-1-phosphotransferase subunit gamma [GNPTG]) on CKD (Fig 3). Of the five proteins, only CTSD had more than 3 *cis-*pQTLs selected as instruments after harmonization. Thus, we used MR-Egger to assess for potential horizontal pleiotropic effect of CTSD on CKD. This resulted in an MR-Egger intercept of 9 x 10^−4^ (*P*-value = 0.24) (S8 Table), suggesting limited risk of directional horizontal pleiotropy.

Importantly, only 9 proteins affected CKD at nominal significance, of which only the effect of GNPTG levels on CKD was significant after multiple testing correction (S8 Table). CKD GWAS was obtained from a prior publication from Wuttke and colleagues [25] (S1 Table), which defined CKD as individuals with eGFR below 60 ml/min/1.73 m^2^. However, there were no genome-wide significant results for CKD in this region (S1 Fig). Of the five proteins with statistically significant effects on kidney function based on MR, the *INHBC* gene was previously implicated in diabetic nephropathy, impaired kidney function, and tissue fibrosis [40,41]. We, therefore, conducted follow-up analyses focusing on this protein. We noted that there were two aptamers targeting INHBC (aptamer 6408-2 and aptamer 15686-49) in the deCODE study. A moderate heterogeneous effect of T2D on INHBC levels was observed for aptamer 6408-2 based on I^2^ statistics (I^2^ = 55%, Q statistics *p*-value = 6.8 x 10^−21^) (S3 Table); therefore, all deCODE results relating to INHBC refer to aptamer 156869 unless otherwise specified.

### 3.3 INHBC follow-up analyses: MR replication in separate cohorts

Four cohorts were used in the replication of the relevant MR results in this study: The UK Biobank [16], EPIC-Norfolk [17], ARIC [19], and Fenland [18] cohorts. The largest sample sizes are from deCODE [15] and the UK Biobank [16] cohort with maximum sample sizes of 35,559 and 34,557 European individuals, respectively. As noted in the original publication, samples from UK Biobank were divided into multiple batches and for the purpose of conducting GWAS, the batch containing COVID-19 longitudinal imaging samples was excluded by the authors [16]. Following these two cohorts, the sample sizes for other cohorts are the 10,708 individuals in the Fenland cohort, the 7,213 participants in the ARIC cohort, and the 2,887 participants in the EPIC-Norfolk cohort (S1 Table).

The estimated MR effects of T2D on circulating INHBC levels were based on 203–206 SNPs across the five cohorts (S9 Table). This effect was replicated in the UK Biobank with the same direction of effect (β= 0.078 [95% CI [0.052, 0.10]), though with a slightly larger effect size (S2A Fig, S9 Table). The estimated MR effect in the EPIC-Norfolk cohort (β = 0.054; 95% confidence interval [−0.011, 0.12]) was similar to what was observed in the deCODE cohort (β = 0.05; 95% confidence interval [0.03, 0.07]), although the 95% confidence interval included the null. The estimated MR effect of T2D on INHBC was directionally consistent, but smaller in the Fenland cohort and the 95% confidence interval also included the null (β = 0.0075; 95% confidence interval [−0.027, 0.042]) (S2A Fig, S9 Table). There was no evidence of weak instrument bias for the MR estimate of T2D on protein level across all cohorts (Minimum F-statistic based on effective sample size: 79) (S9 Table). There was also no evidence of heterogeneous effects among T2D instruments on circulating INHBC levels in the EPIC-Norfolk and the Fenland cohorts (Minimum Q-statistic p-value: 0.14) (S9 Table). Moderate heterogeneity was observed in the UKB cohort based on an I^2^ threshold of 50 (S9 Table).

The estimated MR effects of INHBC on creatinine-based eGFR (eGFR_creatinine_) and BUN were replicated across all four cohorts (deCODE, Fenland, ARIC, and EPIC-Norfolk. p-values between: 1.60 x 10^−4^ and 3.82 x 10^−28^ (S2B Fig, S9 Table). The minimum F-statistic was 426 for the Fenland cohort (S9 Table).

We examined the LD between the INHBC instrument (rs12423654) in deCODE and INHBC instruments obtained from Fenland, ARIC, UKB, and EPIC-Norfolk based on R^2^. We noted that the R^2^ between the instruments was low. The largest R^2^ was 0.43 between the deCODE instrument (rs12423654) and one of the UKB instruments (rs3204635) (S6 Table). This is below the threshold of 0.8 typically used for defining proxy variants in MR analyses, and provides evidence that the results were not cohort specific.

### 3.4 INHBC follow-up analyses: MR replication with cystatin based eGFR

eGFR can be calculated from either creatinine or cystatin. To rule out the possibility that the association of INHBC to eGFR_creatinine_ was due to creatinine production, we performed the same MR analysis with cystatin-based eGFR (eGFR_cystatin_). eGFR_cystatin_ GWAS was obtained from the CKD-GEN consortium. The MR result of INHBC on eGFR_cystatin_ (effect size = −0.002; 95% confidence interval [−0.003, −5.9 x 10^−4^], p-value = 5.1 x 10^−3^) was consistent with the estimated MR effect obtained for eGFR_creatinine_ (effect size = −0.002; 95% confidence interval [−0.003, −0.001]; *p*-value = 3.1 x 10^−6^) (S3 Fig). Similarly, eGFR_cystatin_ does not appear to alter the INHBC level. Collectively, we found that increased INHBC had a consistent negative effect on three kidney function biomarkers (i.e., increased INHBC levels reduce eGFR_creatinine_, eGFR_cystatin_ and increase BUN).

### 3.5 INHBC follow-up analyses: Multivariable MR of BMI, WHR, and T2D on INHBC levels

There was moderate heterogeneity based on an I^2^ threshold of 50 in the estimated causal effects of T2D on INHBC levels in UKB-PPP and deCODE, as measured by Olink and Somalogic aptamer 6408-2, respectively (S10 Table). Aptamer 6408-2 targets an unknown region in the full protein whereas aptamer 15686-49 targets a region specifically within the mature domain in the C-terminus of INHBC. We also confirmed that the Olink platform targets the C-terminus of INHBC. One potential cause of heterogeneous effects is horizontal pleiotropy. As T2D shares genetic determinants with BMI, and WHR with previously estimated genetic correlations of 0.56 and 0.57 respectively [42], we suspected that the horizontal pleiotropic effects of T2D on INHBC may be mediated through one of these related traits. Therefore, we performed multivariable MR to assess the direct effect of each exposure on INHBC level as measured by aptamer 6408-2, 15686-49, or Olink. We found that T2D, BMI, and WHR were each causally linked to increased INHBC levels (S4 Fig). Moreover, we noted that the I^2^ reduced substantially in the MVMR setting (maximum MVMR I^2^ of 28.8 in the UKB-PPP cohort) (S10 Table). This suggested that T2D influences circulating INHBC levels independent of BMI and WHR.

### 3.6 INHBC follow-up analysis: Pairwise conditional and colocalization analyses (PWCoCo)

We performed pairwise conditional and colocalization implemented in PWCoCo v3 on all significant results reported in step 2 MR, namely INHBC on eGFR, and BUN; AGRN on eGFR; CTSD on eGFR, LPO on eGFR and GNPTG on eGFR. PWCoCo is a software package that assesses the probability of shared causal variants within a prespecified region between two traits that can accommodate multiple causal variants [29,43].

**Fig 4:**
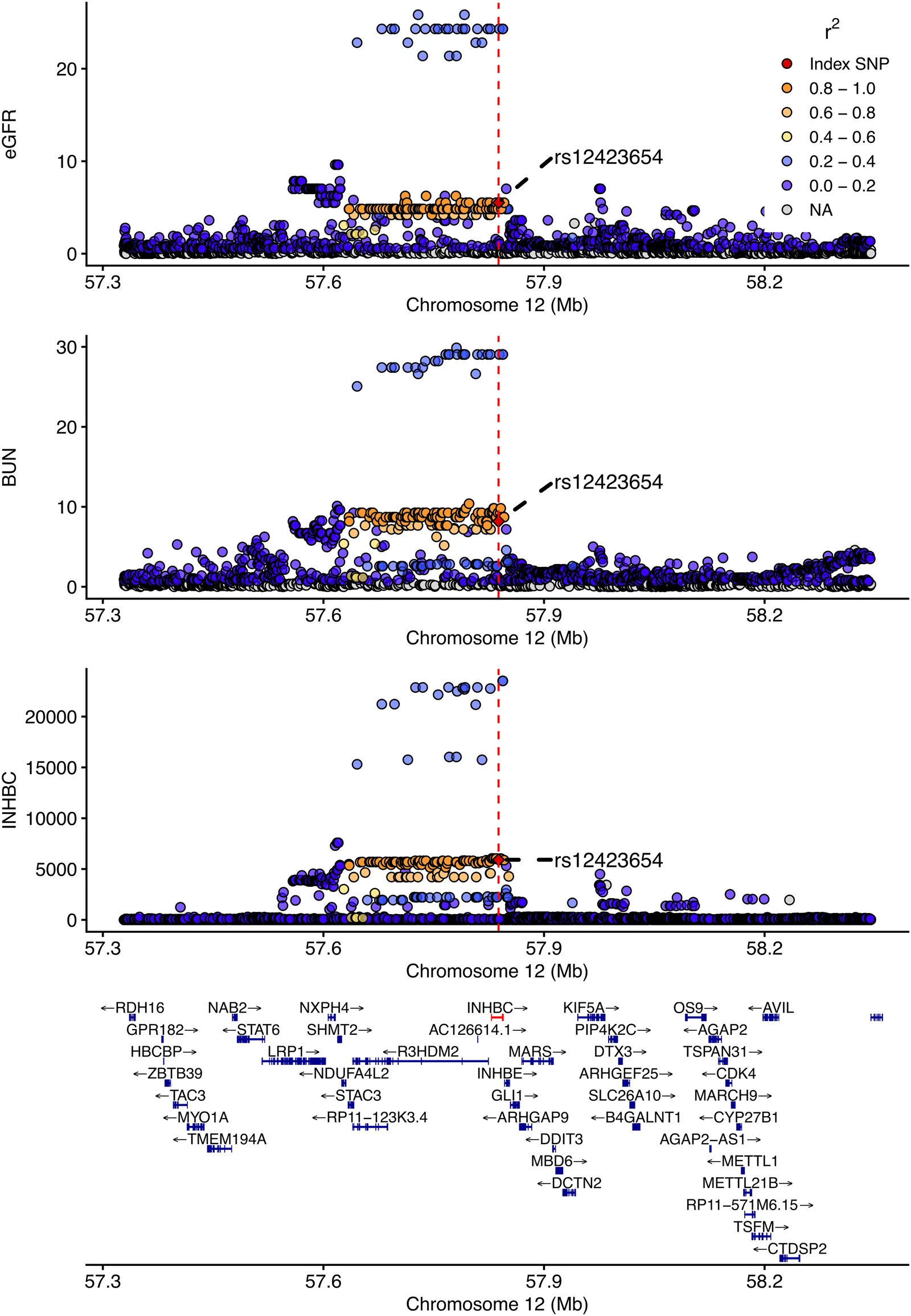
Locuszoom plot of the 1 megabase pair region around the deCODE instrument (rs12423654) for INHBC. The same region for eGFR_creatinine_, and BUN are shown. The red dotted line marks the location of the deCODE instrument (rs12423654). Posterior probability of shared causal variants is 0.98 and 0.96 for BUN and eGFR_creatinine,_ respectively. eGFR: estimated glomerular filtration rate based on serum creatinine, BUN: Blood urea nitrogen.

Focusing on INHBC, within a one megabase pair region around the deCODE genetic instrumental variable (rs12423654), we observed strong colocalization support for shared causal variants with blood urea nitrogen and eGFR_creatinine_ (the H4 posterior probabilities were 0.98 and 0.96 respectively, S11 Table, Fig 4). We also performed the same analysis for a one megabase pair region around the most significant INHBC *cis-*pQTL in UKB (rs3741414). Similarly, with the UK Biobank data, we noted strong colocalization support for shared causal variants with eGFR_creatinine_ and BUN (the H4 posterior probabilities were of 0.92 and 0.98 respectively) (S11 Table, S5 Fig).

### 3.7 INHBC follow-up analysis: Genetic architecture of the nearby region

An important limitation of the MR results is the proximity of INHBC with two nearby genes, namely R3HDM2 and INHBE (Fig 4). We noted strong LD of the deCODE INHBC instrument (rs12423654) with nearby variants (LD R2 ≥ 0.8) (Figs 4, S5). As neither R3HDM2 nor INHBE were measured in the UKB-PPP or the deCODE cohorts, it was impossible to know whether the deCODE instrument is also a *cis-*pQTL for these proteins. Therefore, we obtained eQTL data from GTEx to try to disentangle the effects of rs12423654 on INHBC, INHBE, and R3HDM2.

INHBE eQTL data and the list of proteins for which the deCODE genetic instrument (rs12423654) is an eQTL for were obtained from the GTEx portal V8. The deCODE MR instrument for circulating INHBC protein levels was not an eQTL for INHBE and was not in LD with INHBE eQTLs (maximum R^2^ of the deCODE MR instrument with INHBE eQTLs was 0.19 with rs2242578) (S12 Table). Unlike the INHBE gene, there was strong LD between the deCODE INHBC genetic instrument with variants located within the *R3HDM2* transcript region (Figs 4, S5). The deCODE MR instrument for circulating INHBC protein levels was also an eQTL for both R3HDM2 (p-value = 0.00032, tissue: cells- Cultured fibroblast, Normalized effect size: 0.076) and INHBC (p-value = 3.3 x 10^−5^, tissue: testis, Normalized effect size = −0.24) (S13 Table). In addition to R3HDM2 and INHBC, the deCODE MR instrument was also a *cis-*eQTL for 10 other proteins across 22 tissue types (S13 Table).

We observed that significant *cis-*eQTLs reported by Genotype-Tissue Expression (GTEx) portal had p-values less significant than the genome-wide significance threshold of 5 x 10^−8^ across all tissues (S12 Table). This limited our ability to evaluate the causal effects of INHBE on kidney function through MR using eQTL data. This is because relying on such instruments will likely introduce weak instrument bias, which in the case of two-sample MR will bias the results toward the null. However, the lack of *cis-*eQTL signal for INHBE together with the strong signals observed in eGFR_creatinine_ and BUN within this region would suggest that INHBE is unlikely to influence kidney function through its expression level. Likewise, no *cis-*eQTL reached the genome-wide significance threshold of 5 x 10^−8^ across all tissues in GTEx v10 release.

We also performed colocalization analysis of a 500,000 bp region surrounding the deCODE INHBC instrument locus with liver and whole blood eQTL data for INHBE and R3HDM2. The results showed no evidence of colocalization between INHBC pQTL locus and INHBE or R3HDM2 in either tissue (S14 Table). This suggests that the INHBC pQTL instrument is not an eQTL for INHBE or R3HDM2 in these two tissues.

### 3.8 INHBC follow-up analyses: Observational associations of INHBC and T2D status in UK Biobank

Phase 2 release of the UK Biobank proteomics data by Sun and colleagues [16] was used to assess the link between INHBC and T2D status. We excluded samples from batch 0 (pilot) and batch 7 (COVID-19). T1D and T2D status were defined as ICD-10 code E10 and E11, respectively (S2 Table), and only prevalent T1D or T2D cases were kept (see Methods for further details). In addition, as INHBC is predominantly produced by the liver, we removed individuals with liver diseases to avoid the confounding effects of liver disease on INHBC levels. Liver diseases were defined based on 8 ICD-10 codes (S2 Table, see Methods for further details). Consistent with MR results, individuals with T2D had higher levels of INHBC at baseline than individuals without T2D (*T* test *p*-value = 2.3 x 10^−62^, S6A Fig). T2D was also significantly positively associated with INHBC level after accounting for age, genetically determined sex, ancestry, and BMI in a multivariable linear regression model (S6B Fig).

### 3.9 INHBC follow-up analyses: Observational associations of INHBC and kidney disease incidence in UK Biobank

Time-to-event analysis was used to evaluate the association of INHBC levels with future kidney disease diagnosis among participants in the UKB-PPP. Kidney diseases were defined based on 18 ICD-10 and 20 OPCS-4 codes (S2 Table). Only individuals whose earliest diagnosis date was after the blood draw date (i.e., incident kidney cases) were kept. Same as before, only prevalent T2D cases were kept and individuals with liver disease, as defined above, were removed (see Methods for more details). In total, 37,854 individuals were used for all observational analyses, of which there were 582 prevalent T2D cases, 43 prevalent T1D cases, and 2,128 incident kidney diseases (S15 Table).

**Fig 5:**
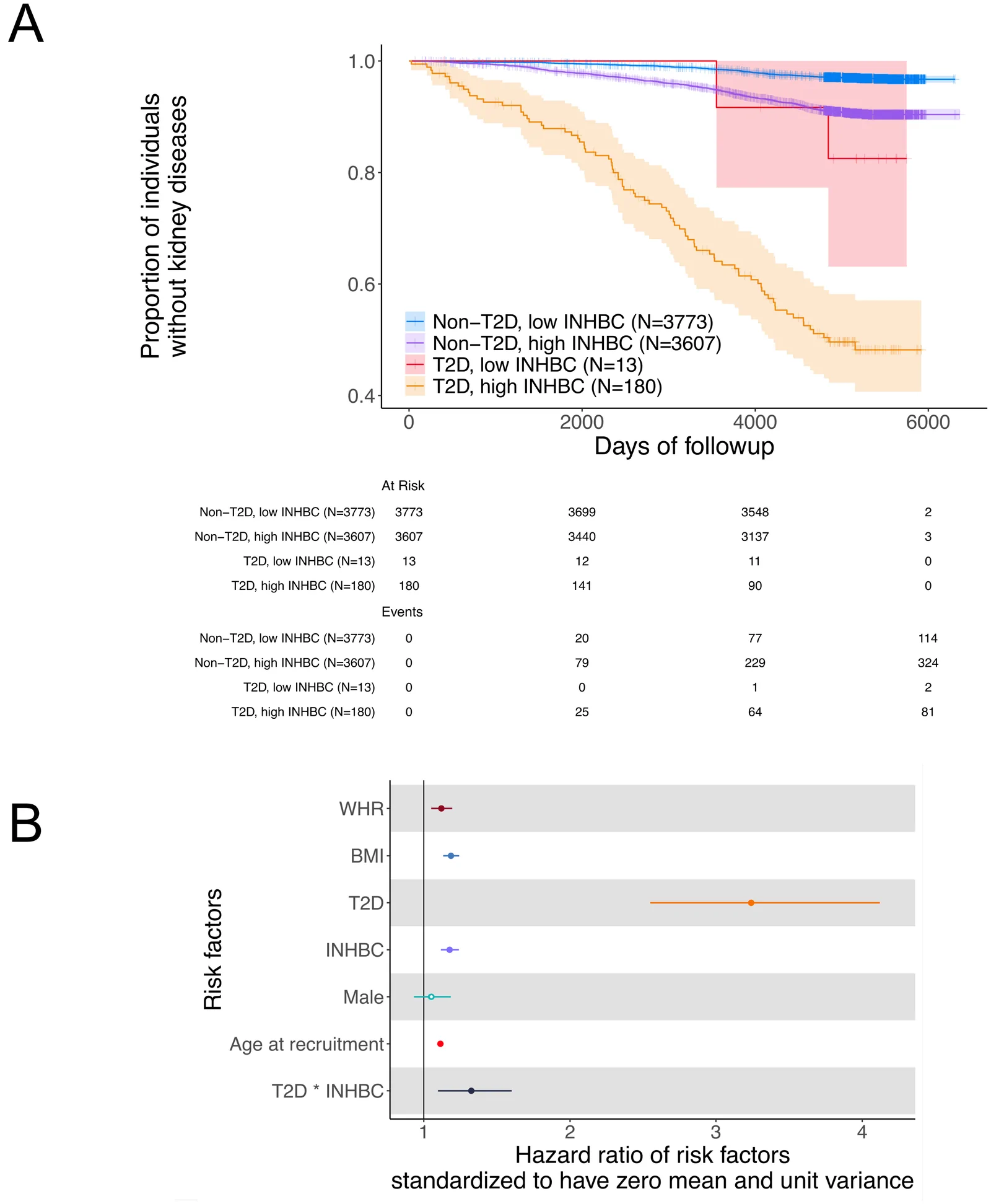
Time-to-event analysis demonstrating the association of serum INHBC levels with kidney disease diagnosis. **A)** Kaplan-Meier curves comparing risk of developing kidney diseases among four groups of individuals: Individuals without T2D with low circulating levels of INHBC (blue), high circulating levels of INHBC (purple) and individuals with T2D with low circulating levels of INHBC (red) and high circulating levels of INHBC levels (orange). High and low circulating levels were determined based on the top and lower 10th percentile, respectively. Ninety-five% confidence interval are shown. **B)** Effect size from multivariable Cox proportional hazard models of various risk factors on kidney disease risk. WHR, BMI, and INHBC levels were standardized to have unit variance and zero mean. T2D and male were coded as binary where 1 represents prevalent T2D cases and males, 0 represents the absence of T2D diagnosis and females respectively. WHR: Waist to hip ratio, BMI: Body mass index, T2D: Type 2 diabetes, INHBC: Inhibin beta C-chain.

KM curves plot the proportion of individuals without kidney diseases as a function of days of follow-up. We found that individuals with T2D had a higher risk of developing kidney diseases than individuals without T2D (Fig 5A). Consistent with the MR results, the majority of the individuals with T2D before blood draw had higher INHBC levels as evidenced by the wider confidence interval observed in the T2D, low INHBC group (Fig 5A). Also consistent with the MR results, individuals with higher levels of INHBC had a higher risk of developing kidney diseases.

To assess the independent contribution to kidney disease risk of relevant covariates, a multivariable Cox proportional hazard model with WHR, BMI, T2D diagnosis status, circulating INHBC levels, genetically determined sex, age and the interaction between T2D diagnosis and circulating INHBC levels was used. Multivariable Cox regression showed that increased WHR, BMI, INHBC level, and T2D were all associated with increased risk of kidney diseases (Fig 5B, S16 Table). Males also had an increased risk of developing kidney diseases compared to females though this was not statistically significant (hazard ratio = 1.05; 95% confidence interval [0.93,1.18]). There was a positive interaction between T2D and INHBC levels (hazard ratio = 1.3; 95% confidence interval [1.1, 1.6]), which suggested that having T2D increases the negative effects of INHBC levels on kidney function measures (S16 Table). However, we note that unadjusted confounding factors is an important limitation of this analysis.

### 3.10 INHBC follow-up analyses: Observational mediation analysis

We conducted mediation analysis for the effect of T2D on circulating INHBC level and in turn, circulating INHBC level on kidney disease status using mediation 4.5.1 R package. Using the same set of samples as the time-to-event analyses, for T2D, we included only those diagnosed before the blood draw date and for kidney disease, we restricted to only those diagnosed after the blood draw date. We further included genetically predicted sex, smoking status, UK Biobank assessment centre, BMI, systolic blood pressure and alcohol intake frequency as covariates. Confidence intervals were estimated with 1,000 bootstrap replications. We noted that circulating INHBC level mediated approximately 1.3% (95% confidence interval [0.85%, 1.9%]) of the association between prevalent T2D status and incident kidney disease.

### 3.11 Comparison of T2D and T1D: Mendelian randomization study

CKD is a common complication for both T2D and T1D patients. These two diseases have distinct pathologies and therefore may have different causal mechanisms for CKD. We next wanted to determine whether INHBC level is a common mediator for CKD among these two diseases. To do so, we tested whether T1D risk was causally linked to changes in INHBC levels. A recent T1D GWAS of European ancestry with 18,942 cases and 501,638 controls was used [44] (S1 Table).

**Fig 6:**
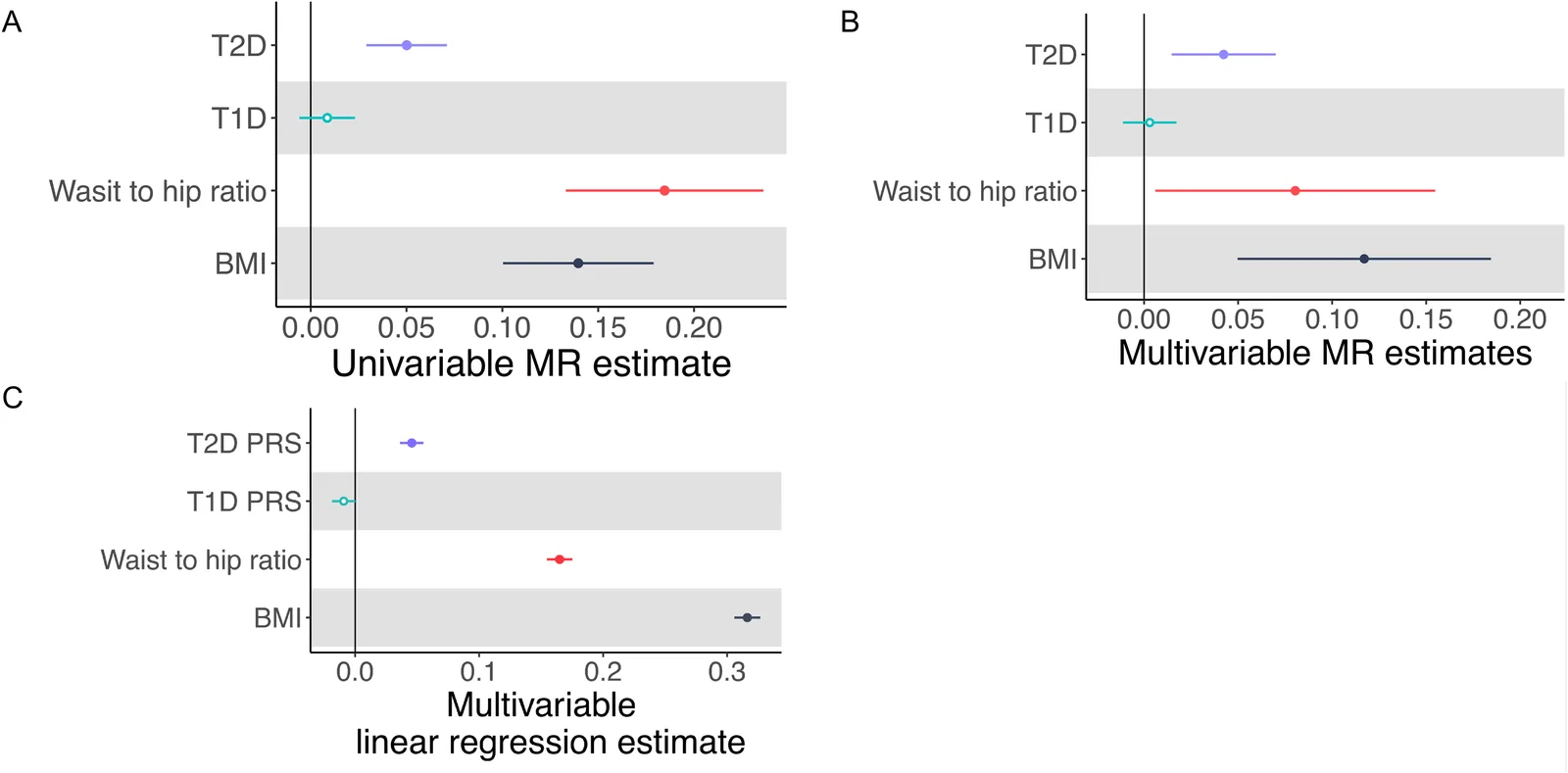
Two-sample mendelian randomization and linear regression results comparing the estimated effects of susceptibility to T2D and T1D on circulating INHBC levels. **A)** Univariable two-sample MR results of the estimated MR effect of susceptibility to T2D, T1D, waist to hip ratio (WHR), and body mass index (BMI) on circulating INHBC levels in deCODE). **B)** Multivariable two-sample MR results of the estimated MR effect of susceptibility to T2D, T1D, WHR and BMI on circulating INHBC levels in deCODE. **C)** Multivariable linear regression of the association of T2D polygenic risk score (PRS), T1D PRS, WHR and BMI with circulating INHBC levels in UKB. T1D and T2D are in log odds ratio. T1D PRS, T2D PRS, WHR, BMI, and INHBC levels were all standardized to have zero mean and unit variance for the linear regression analyses. WHR, BMI, and INHBC levels were inverse rank normal transformed for the MR analyses. T2D: Type 2 diabetes, T1D: Type 1 diabetes, PRS: Polygenic risk scores.

We noted that in univariable MR analysis, genetic susceptibility to T1D did not significantly alter the levels of INHBC (β = 8.6 x 10^−3^, p = 0.24) (Fig 6A). Similar results were seen in multivariable MR accounting for the effects of T1D (β = 3 x 10^−3^, p = 0.68, F-statistic = 12.9), T2D (β = 0.04, p = 2.8 x 10^−3^, F-statistic = 20.6), BMI (β = 0.12, p = 6.6 x 10^−4^, F-statistic = 46.2), and WHR (β = 0.08, p = 0.03, F-statistic = 32.1) (Fig 6B).

### 3.12 Comparison of T2D and T1D: Observational study in UK Biobank

To determine observationally whether the effects of T1D and T2D on INHBC level were consistent with MR findings, we performed association analysis among the same samples that were used for the time-to-event analysis (S2 Table). For the association analysis, we used T2D and T1D PRS released by Thompson and colleagues [45]. We found a negative, though not statistically significant association between T1D PRS and INHBC levels whereas T2D PRS was positively and significantly associated with INHBC levels (Fig 6C). This suggested that observationally, a high genetically predicted risk of T2D was associated with higher levels of circulating INHBC. WHR and BMI were both positively associated with circulating levels of INHBC.

## 4 Discussion

To understand how T2D influences kidney disease risk beyond standard MR screening, we used MR in a two step process to identify proteins that partially mediate the effect of T2D on kidney function. Such a question is therefore distinct from identifying all proteins that influence eGFR. We first identified 71 proteins whose levels were altered by increased genetic susceptibility to T2D. We found that increased genetic susceptibility to T2D was associated with an increase in circulating inhibin beta C chain (INHBC) protein levels and that higher circulating levels of INHBC led to reduced renal function. Consistent with MR findings, observational analyses indicated that increased circulating INHBC level was a risk factor for kidney disease independent of BMI, WHR, age, and sex. Collectively, it suggested that INHBC and its associated pathways may contribute to the progression of CKD amongst individuals with T2D.

Previous studies have shown that increased levels of transforming growth factor β (*TGF-*β) and activin A are associated with an increased risk of kidney diseases and that activin A neutralization can inhibit fibrosis induced by unilateral ureteral obstruction [46,47]. INHBC is in the same superfamily of proteins as activin A. INHBC was also implicated in kidney diseases, and diabetic nephropathy based on transcriptome/proteome-wide association studies, MR analysis, and animal models [34–36,40]. Goebel and colleagues have previously shown that activin C and activin AC can stimulate SMAD2/3 proteins, which are known to be involved in tissue fibrosis [48,49], via the type I receptors [41]. Taken together, it suggests that INHBC inhibition may also have reno-protective effects. Despite existing studies, causality between T2D and kidney diseases via circulating INHBC protein level was not previously established. Therefore, we conducted follow-up analyses focusing on INHBC based on the likely role it plays in mediating the effect of T2D on DKD.

Our preliminary findings arose from the genetic determinants of INHBC, as assessed in the deCODe cohort, but the estimated MR effects of INHBC on eGFR_creatinine_ and BUN were also replicated in the Fenland, UK Biobank, EPIC-Norfolk, and the ARIC cohorts. Different genetic instruments for INHBC were selected for each cohort and were not in LD with the INHBC instrument identified from the deCODE study; the maximum R^2^ was 0.43 between one of the UK Biobank instruments (rs3204635) and the deCODE instrument (rs12423654). Furthermore, while some cohorts use the SomaLogic platform others use the Olink platform. Despite the differences in proteomic platform and population, these results were replicated. This provided confidence that the MR results between INHBC and these kidney function biomarkers were not due to cohort-specific population structure, or peculiarities of the SomaLogic or Olink platforms.

Although moderate heterogeneous effects were observed for the MR results of T2D on INHBC based on the UK Biobank data and aptamer 6408-2 in deCODE, no heterogeneous effects (based on an I^2^ threshold of 50 or a Q-statistic *p*-value > 0.05) were observed after adjusting for BMI and WHR in a multivariable MR setting. The effects of T2D on INHBC remained significant in MVMR. This suggested that the estimated MR effect of T2D on INHBC was not entirely due to the shared genetic effects between T2D, BMI, and WHR.

The estimated MR effect of T2D on INHBC level was supported by observational data within the UK Biobank cohort. Of the 37,854 European individuals who underwent proteomics study, 1.5% (*N* = 582) of them were diagnosed with T2D based on ICD-10 code E11 before the blood draw date (i.e., prevalent T2D cases). Individuals with T2D had significantly higher levels of circulating INHBC levels than individuals without T2D at baseline (*t* test *p*-value of 2.3 x 10^−62^). This is consistent with the MR results where increased genetic susceptibility to T2D was associated with increased INHBC levels. T2D remained significantly associated with INHBC levels after accounting for genetically predicted sex, age, ancestry and BMI based on a linear regression model (β = 0.20, *p*-value = 2.6 x 10^−7^). Based on observational, cross-sectional data, we noted that circulating INHBC level mediated 1.3% (95% confidence interval [0.85%, 1.9%]) of the association between T2D and kidney disease diagnosis consistent with the causal pathway suggested based on MR analyses. This represents the extent to which INHBC mediates the effect between T2D and kidney disease after controlling for other variables based on observational data. Given the polygenic nature of T2D and we are examining the proportion of effects mediated by a single protein, a modest effect size is to be expected. However, this result should be interpreted with caution due unadjusted confounders and the fundamental differences between observational analyses and clinical pharmaceutical interventions.

We also performed time-to-event analyses to assess the association of INHBC levels and kidney disease diagnosis among the 37,854 European individuals in the UK Biobank. We noticed that, as expected, individuals with T2D were at a higher risk of developing kidney diseases compared to individuals without T2D. In the multivariable Cox proportional hazard model, the hazard ratio for developing kidney disease associated with INHBC levels was 1.17 (95% CI [1.12, 1.23]) per SD increase in INHBC level. There was also a positive interaction between T2D status and INHBC with an associated hazard ratio of 1.32 (95% CI [1.1, 1.6]). This suggested that the deleterious effect of circulating INHBC levels on kidney disease risk was stronger among individuals living with T2D than nondiabetics.

Despite fundamental differences in the disease pathology of T2D and T1D patients, CKD occurs in both. We, therefore, tested whether T2D and T1D influence INHBC levels similarly. Both univariable and multivariable MR analyses show that T1D had a much smaller estimated MR effect, if any, on INHBC levels than T2D. Observationally, we noted a negative, but not statistically significant association between T1D PRS with INHBC levels whereas T2D PRS was positively and significantly associated with INHBC levels. This suggested that INHBC may have a different role in CKD among T2D and T1D patients.

There are important limitations to this study. Firstly, this study was limited by the protein assayed in the two proteomics platforms (SomaLogic and Olink) and the availability of *cis-*pQTL. Of the 4,907 proteins assayed in deCODE, only 1,721 proteins have *cis-*pQTLs after filtering (see Methods for further detail) thus limiting our ability to fully explore the effects of the proteome on CKD. For instance, INHBC may exist as a homodier named Activin C or as a heterodimer with other inhibins. The current study design does not allow us to differentiate the roles between homo- and hetero-dimers of INHBC. Furthermore, two genes near *INHBC*, namely *R3HDM2* and *INHBE*, were not measured on the proteomic platforms. The proximity of *R3HDM2* and *INHBE* with *INHBC* made it difficult to disentangle the causal effects of the three genes on kidney function based on genetic data as we could not determine if the INHBC *cis-*pQTL was shared across the three genes. This is particularly important, given recent randomized control trial (RCT) data and human genetics data showing beneficial effects of INHBE inhibition on obesity-related traits [50,51]. We, therefore, had to rely on other data. Reassuringly, the deCODE instrument used for INHBC (rs12423654) was not an eQTL for INHBE and was not in strong LD with INHBE eQTLs. In addition, INHBC is predominantly produced by the liver, and there was no evidence of colocalization between the deCODE instrument and either INHBE or R3HDM2 expression in liver or whole blood tissue. Therefore, it is not unreasonable to assume that the effect of the INHBC instrument on T2D through INHBE was small. However, the deCODE instrument for INHBC is an eQTL for both INHBC and R3HDM2 in the testis and culture fibroblast tissue, respectively; therefore, neither pQTLs nor eQTLs were sufficient to unambiguously disentangle the effects of the two proteins on kidney function. Although, based on prior evidence, we suspected that INHBC plays a role in the development of CKD, we cannot rule out potential horizontal pleiotropic effects without additional GWAS data of R3HDM2. We can, however, be reasonably certain that the *INHBC*-*R3HDM2* region is associated with eGFR_creatinine_ and BUN based on the strong GWAS signal in this region across both traits (Figs 4, S5). Secondly, the MR result of T2D on INHBC was not replicated in the Fenland cohort. As both the Fenland cohort and UK Biobank used the Olink platform, it is unlikely to have platform-specific effects. Further studies are required to determine the cohort-specific variations or other factors that could lead to this difference. Third, we note that pleiotropic robust MR methods, such as MR-Egger, require many variants to provide an accurate estimate of pleiotropic effect; therefore, estimates in the second stage of MR (i.e., protein on disease) can be variable due to a limited number of *cis-*pQTLs. In part to address this limitation, we conducted colocalization analyses and saw strong colocalization between relevant exposure outcome pairs. Fourth, we note that T2D susceptibility was not associated with changes in circulating INHBB levels based on MR analyses (S3 Table). However, we note that INHBB, and INHBA are likely regulated differently than INHBC as previously shown [41]. Consequently, further studies are required to further elucidate the role of INHBC and the TGF-β pathway in diabetic kidney diseases. Fifth, we note that there are important caveats in interpreting MR results with binary traits as exposures [52]. Genetic signals associated with diseases will likely be associated with other causal factors or comorbidities which, in the context of MR, may lead to horizontal pleiotropic effects. While we cannot fully rule out all horizontal pleiotropic effects, we have attempted to account for this through multivariable MR. Another caveat is that the genetic variants may alter the disease liability without changing disease status (e.g., genetic variants may increase an individual’s HbA1c level substantially, but the individual may remain below the threshold for diabetes diagnosis). However, it was previously shown that while this will alter the magnitude of the MR estimates, it should not reverse the signs of the estimates [53]. Thus, while the magnitude of the MR estimates presented in step 1 may be biased due to the use of a binary trait as the exposure, the two-step approach should still allow us to infer the correct causal direction. Lastly, we note that the datasets we used in this study were population based and not focused on diabetic kidney disease. Therefore, follow-up studies focusing on individuals with diabetic kidney disease among individuals with diabetes will be required to validate the role of INHBC in diabetic kidney disease and our results remain only relevant to kidney disease in general.

In this study, we have, through the integration of proteomics and genomics data, identified five proteins that likely mediate the effects of T2D on kidney function. In particular, INHBC was the most likely candidate supported by consistent adverse effects on kidney function as assessed by eGFR_creatinine_, and BUN along with prior studies. The estimated negative MR effects of increased INHBC levels on eGFR_creatinine_ and BUN were replicated in the Fenland, UK Biobank, EPIC-Norfolk, and ARIC cohorts. Observational data within the UK Biobank cohort also supported the association between T2D, INHBC, and kidney diseases. Collectively, this suggested that INHBC and its associated pathways may contribute to the development of CKD.

## Supporting information

S1 Text Supplementary text. (DOCX)

S1 Table GWAS sources and sample size information for traits/diseases used in this study. (XLSX)

S2 Table ICD-10 and OPCS-4 codes used for disease definitions. (XLSX)

S3 Table Two-sample MR results of the estimated causal effects of T2D susceptibility on protein levels. Protein GWAS were obtained from deCODE.
(XLSX)

S4 Table Genetic instruments of nonprotein traits used in this study. Genome positions are in GRCh37.
(XLSX)

S5 Table Genetic instruments obtained from deCODE for the 5 proteins influenced by T2D susceptibility that in turn affects kidney function. SNP positions are reported in GRCh37.
(XLSX)

S6 Table Linkage disequilibrium R2 between deCODE INHBC instruments and INHBC instruments from other cohorts. (XLSX)

S7 Table Steiger test results for the relevant MR results as reported by the TwoSampleMR package. (XLSX)

S8 Table Two-sample MR results of the 71 proteins influenced by T2D susceptibility on four kidney phenotypes. Protein GWAS were obtained from deCODE.
(XLSX)

S9 Table MR results of the estimated casual effect of T2D on INHBC and INHBC on eGFR/BUN across the five proteomics cohorts. (XLSX)

S10 Table Heterogeniety and F-statistics for multivariable MR and MR of T2D on INHBC levels. (XLSX)

S11 Table PWCoCo outputs in a 500,000 base pair region on either side of the *cis-*pQTL for significant protein to kidney trait MR as defined in Fig 2. (XLSX)

S12 Table INHBE eQTLs obtained from the GTEx portal (V8) and R2 with the deCODE IV. (XLSX)

S13 Table Genes for which the deCODE INHBC instrument (rs12423654) is an eQTL for obtained from the GTEx portal. (XLSX)

S14 Table PWCoCo analysis of INHBC pQTL region with eQTL of INHBE and R3HDM2 in whole blood and liver tissues. (XLSX)

S15 Table Demographic information of individuals used in observational analyses. (XLSX)

S16 Table Multivaribale Cox proporitional hazard regression results. (XLSX)

S17 Table Mediation analysis summary. (XLSX)

S1 Checklist STROBE-MR checklist. (DOCX)

S1 Fig Locuszoom plot of a 1 megabase pair region around the deCODE instrument (rs9923746) for GNPTG. The same region for chronic kidney disease (CKD) is shown. The red dotted line marks the location of the deCODE instrument (rs9923746).
(TIFF)

S2 Fig Results of the two-sample MR replication across four additional cohorts (UKB: UK Biobank pharma proteomics project, ARIC: Atherosclerosis Risk in Communities, EPIC-Norfolk, and Fenland). A) The estimated MR effects of genetic susceptibility to T2D on INHBC levels. B) The estimated MR effects of INHBC levels on kidney function. Unadjusted p-values and 95% confidence intervals are shown. Units for T2D, blood urea nitrogen, and estimated glomerular filtration rate are log odds ratio, ln(mg/dL), and ln(ml/min/1.73 m^2^), respectively. eGFR: estimated glomerular filtration rate. BUN: Blood urea nitrogen.
(TIFF)

S3 Fig Two-sample MR results of the estimated MR effects of INHBC on cystatin-based eGFR (eGFR_cystatin_) and creatinine-based eGFR (eGFR_creatinine_). Unadjusted *p*-values and 95% confidence intervals are plotted. The units for eGFR (creatinine and cystatin based) are ln(ml/min/1.73 m^2^). INHBC levels were inverse normal rank transformed. eGFR: estimated glomerular filtration rate.
(TIFF)

S4 Fig Multivariable and univariable two-sample mendelian randomization results of the estimated MR effects of susceptibility to type 2 diabetes (T2D), body mass index (BMI) and waist-to-hip ratio (WHR) on INHBC levels. The two aptamers for INHBC used in deCODE were 15686-49 and 6408-2. Units for T2D are in log odds ratio. INHBC levels, BMI and WHR were inverse normal rank transformed.
(TIFF)

S5 Fig Locuszoom plot of the 1 megabase pair region around the most significant UKB instrument (rs3741414) for INHBC. The same region for eGFR_creatinine_ and BUN are shown. The red dotted line marks the location of the UKB instrument (rs3741414). eGFR: estimated glomerular filtration rate. BUN: Blood urea nitrogen.
(TIFF)

S6 Fig Observational association of INHBC levels as measured by Olink anti-body assay and prevalent T2D status in UK Biobank cohort. A) Distribution of INHBC levels among individuals with and without T2D (prevalent cases only). B) Association of INHBC levels (standardized to have zero mean and unit variance) with prevalent T2D status, age, and genetically determined sex as assessed by multivariable linear regression. NPX: Normalized protein expression as reported by UKB-PPP.
(TIFF)

## Abbreviations

AICc: Akaike information criterion
ARIC: Atherosclerosis Risk in Communities
BMI: body mass index
BUN: blood urea nitrogen
CKD: chronic kidney disease
CKD-EPI: Chronic Kidney Disease Epidemiology Collaboration
COVID-19: Coronavirus disease 2019
eGFR: glomerular filtration rate
EPIC: European Prospective Investigation of Cancer
GWAS: genome-wide association studies
GTEx: Genotype-Tissue Expression
ICD-10: International Classification of Diseases 10th revision
INHBC: inhibin beta C-chain level
KM: Kaplan-Meier
LD: linkage disequilibrium
MR: Mendelian randomization
MVMR: multivariable MR;
OPCS-4: Office of Population Censuses and Surveys Classification of Interventions and Procedures version 4
pQTL: Protein quantitative trait loci
PRS: polygenic risk score
R3HDM2: R3H domain-containing protein 2
RAS: Renin-angiotensin system
RCT: randomized control trial
SD: standard deviation
SHBG: sex hormone-binding globulin
SNPs: single nucleotide polymorphisms
SuSiE: sum of single-effects regression; T1D, type 1 diabetes
T2D: type 2 diabetes
TGF-𝛽: transforming growth factor 𝛽
uACR: urinary albumin-to-creatinine
WHR: Waist-to-hip ratio.
