# Supplementary material for: Protein mediators of chronic kidney disease in Type 2 diabetes: A mendelian randomization study
Source: PLoS Med. 2026 Sep 11;23(9):e1004802. doi: 10.1371/journal.pmed.1004802 (PMC13568495; doi:10.1371/journal.pmed.1004802)
Supplement: Supplementary file 1 [file pmed.1004802.s001.docx]

**Protein mediators of chronic kidney disease in Type 2 diabetes : a mendelian randomization study**

Kevin Y. H. Liang^1,2^, Thomas M. Zheng^1,3^, Dandan Tan^1,3^, Takayoshi Sasako^1,4,5,6^, Yann Ilboudo^7,8^, Yiheng Chen^9^, Guillaume Butler-Laporte^1,10,11^, Satoshi Yoshiji^1,12,13^, J. Brent Richards^1,2,12, 14, 15, 16^

1 Lady Davis Institute for Medical Research, Jewish General Hospital, Montréal, Québec, Canada

2 5 Prime Sciences Inc, Montréal, Québec, Canada

3 Quantitative Life Sciences, McGill University, Montréal, Québec, Canada

4 Tanaka Diabetes Clinic Omiya, Saitama, Japan

5 Okachimachi Ohisama Clinic, Tokyo, Japan

6 Department of Diabetes, Metabolism and Endocrinology, Ichikawa Medical Center, International University of Health and Welfare, Chiba, Japan

7 Channing Division of Network Medicine, Department of Medicine, Brigham and Women’s Hospital

8 Harvard Medical School, Boston, MA, USA

9 Precision Healthcare University Research Institute (PHURI), Queen Mary University of London, London, UK

10 Centre for Human Genetics, University of Oxford, Oxford, UK

11 Division of Infectious Diseases, McGill University Health Centre, Montréal, Québec, Canada

12 Department of Human Genetics, McGill University, Montréal, Québec, Canada

13 Programs in Metabolism and Medical & Population Genetics, The Broad Institute of MIT and Harvard, Cambridge, MA, USA

14 Department of Epidemiology, Biostatistics and Occupational Health, McGill University, Montréal, Québec, Canada

15 Department of Twin Research, King's College London, London, UK.

16 Department of Medicine, McGill University, Montréal, Quebec, Canada

Corresponding author: J. Brent Richards. [brent.richards@mcgill.ca](mailto:brent.richards@mcgill.ca)

# 1 Supplementary Text

## 1.1 MR sensitivity analysis: Assessment of alternative MR effect estimates and reverse causation

1 We used MR-Egger, weighted median, weighted mode, and simple mode to estimate the causal effects of T2D on circulating protein levels. These methods can tolerate violation of the horizontal pleiotropy assumptions to different degrees, though often with reduced statistical power. In all cases, the estimated MR effects of T2D on the five proteins (i.e., AGRN, CTSD, GNPTG, INHBC, and LPO) were in the same direction as the inverse variance weighted estimate (i.e., increased susceptibility to T2D increased protein levels) (Table S2). Likewise, for Step 2 MR, when sufficient instruments were present, we also estimated the MR effects using MR-Egger, weighted median, weighted mode, and simple mode. Of the five proteins, only the effects of CTSD on eGFR had sufficient instrumental variables to perform the other MR estimates. The estimated MR effects were in the same direction as the inverse variance weighted estimate (Table S5).
2 Reverse causation was assessed using two methods: Bi-directional MR, and the Steiger test. For step 1, after Bonferroni correction for each direction (p-value of 0.05/1,721 = 2.9 x 10^-5^ and 0.05/1,517=3.3 x 10^-5^ for forward and reverse direction respectively), we identified 71 proteins whose levels were likely influenced by genetic susceptibility to T2D and in the correct causal direction. For step 2, after Bonferroni correction for each direction (p-value threshold: 0.05/278=1.8 x 10^-4^ and 0.05/284=1.8 x 10^-4^ for forward and reverse respectively), 6 significant MR results were likely to be in the correct causal direction (i.e., protein levels influence kidney functions). This included the effects of 4 proteins (AGRN, CTSD, INHBC, and LPO) on two kidney function biomarkers (eGFR, BUN) and one protein (GNPTG) on chronic kidney disease. We then performed Steiger tests for the 6 significant MR results and the effects of T2D on the 5 proteins identified in Step 2. The Steiger test requires disease prevalence for case/control studies and sample size. We used the effective sample size and assumed the prevalence of T2D and CKD among individuals of European ancestries to be 9.2% and 12.8% respectively as previously reported[1,2], the direction of effects of all results was correct (Table S7).
3 Furthermore, we noted that only one of the 203 T2D genetic instruments was within 10 megabase pairs on either side of the INHBC transcript region (rs2258238: p-value 5.14 x 10^-22^). MR analysis using the remaining 202 T2D genetic instruments on INHBC had similar results as prior estimates (Number of SNPs: 202, MR-IVW beta: 0.05, MR-IVW se: 0.011, MR-IVW p-value: 3.28 x 10^-6^, F-stat: 79). We also performed variant-level Steiger filtering using the *steiger_filtering()* function implemented in TwoSampleMR v0.5.7 to further validate the direction of the estimated MR effects of T2D on INHBC levels. After Steiger filtering, 201 genetic variants remained for MR analysis as opposed to the 203 originally used. The cumulative F-statistic for the 201 genetic variants was 79. The MR estimate was 0.04 (95% CI: 0.025 – 0.063). This is also consistent with the prior estimate of 0.05 (95% CI: 0.029 – 0.071).

## 1.2 MR sensitivity analysis: Assess for proteins with shared cis-pQTLs used in Step 2

1 For the 5 proteins identified in step 2 that were influenced by T2D susceptibility and significantly affected kidney functions, we checked whether the cis-pQTLs used as instrumental variables were unique to each protein. Across the 5 proteins, 19 cis-pQTLs were used as instruments ranging from 7 cis-pQTLs for AGRN to 1 cis-pQTL for INHBC. All pQTLs were cis-pQTLs for only one protein (Table S16). One cis-pQTL for LPO, rs7219860, was also a trans-pQTL for VAMP7.

## 1.3 MR sensitivity analysis: Assess maximum relative bias due to sample overlap

1 The relative bias due to sample overlap can be interpreted as the bias in the MR estimate relative to the bias in the observational estimate. It is a function of the proportion of sample overlap and the strength of association (smaller sample overlap and/or stronger instrument strength leads to less bias)[3]. Assuming maximum overlap and using the F-statistic for INHBC and T2D, we can compute the maximum relative bias of the MR analyses used to assess the causal effect of T2D on INHBC (step 1) and INHBC on kidney traits (step 2).
2 The F-statistic for T2D was 79.8 with 80,154 cases and 853,816 controls (effective sample size of 14,6550). The maximum sample size for deCODE was 35,559. The maximum proportion of overlap was therefore 0.24 (35559/146550) with a maximum relative bias of 0.5% for step 1 MR (i.e., T2D on protein). The F-statistic for INHBC was 1374. The minimum sample size for the 3 kidney phenotypes altered by INHBC level (i.e., CKD, eGFR, and BUN) was CKD with 41,395 cases and 439,303 controls (effective sample size of 75,661) (Table S1). The maximum proportion of sample overlap was therefore 0.47 (i.e., 35,559/75,661) with a maximum relative bias of 0.05%. Overall, the maximum relative bias of relevant MR analyses was found to be low.

# References

1.	Sun H, Saeedi P, Karuranga S, Pinkepank M, Ogurtsova K, Duncan BB, et al. IDF Diabetes Atlas: Global, regional and country-level diabetes prevalence estimates for 2021 and projections for 2045. Diabetes Res Clin Pract. 2022;183: 109119. doi:10.1016/j.diabres.2021.109119

2.	Bello AK, Okpechi IG, Levin A, Ye F, Damster S, Arruebo S, et al. An update on the global disparities in kidney disease burden and care across world countries and regions. Lancet Glob Health. 2024;12: e382–e395. doi:10.1016/S2214-109X(23)00570-3

3.	Burgess S, Davies NM, Thompson SG. Bias due to participant overlap in two‐sample Mendelian randomization. Genet Epidemiol. 2016;40: 597–608. doi:10.1002/gepi.21998
