# Supplementary material for: Protein mediators of chronic kidney disease in Type 2 diabetes: A mendelian randomization study
Source: PLoS Med. 2026 Sep 11;23(9):e1004802. doi: 10.1371/journal.pmed.1004802 (PMC13568495; doi:10.1371/journal.pmed.1004802)
Supplement: Supplementary file 2 [file pmed.1004802.s019.docx]

**STROBE-MR checklist of recommended items to address in reports of Mendelian randomization studies**^1^ ^2^

| **Item No.** | **Section** | **Checklist item** | **Page No.** | | **Relevant text from manuscript** |
| --- | --- | --- | --- | --- | --- |
| 1 | **TITLE and ABSTRACT** | Indicate Mendelian randomization (MR) as the study’s design in the title and/or the abstract if that is a main purpose of the study | Abstract, paragraphs 1-3 |  | |
|  | **INTRODUCTION** |  |  |  | |
| 2 | **Background** | Explain the scientific background and rationale for the reported study. What is the exposure? Is a potential causal relationship between exposure and outcome plausible? Justify why MR is a helpful method to address the study question | Section 1, paragraphs 3-5 | This is discussed in the Introduction. | |
| 3 | **Objectives** | State specific objectives clearly, including pre-specified causal hypotheses (if any). State that MR is a method that, under specific assumptions, intends to estimate causal effects | Section 1, paragraphs 3-5 | This is stated in the Introduction | |
|  | **METHODS** |  |  |  | |
| 4 | **Study design and data sources** | Present key elements of the study design early in the article. Consider including a table listing sources of data for all phases of the study. For each data source contributing to the analysis, describe the following: | N/A | Sources of GWAS are listed in Supplementary Table 1 | |
|  | a) | Setting: Describe the study design and the underlying population, if possible. Describe the setting, locations, and relevant dates, including periods of recruitment, exposure, follow-up, and data collection, when available. | Section 2.1, paragraphs 1  Section 2.2.1, paragraphs 1-6 | Summary statistics are from publicly available sources.  They are listed in Supplementary table 1  Summary level information for observational analyses are listed in Supplementary table 15.  It is described in the Methods | |
|  | b) | Participants: Give the eligibility criteria, and the sources and methods of selection of participants. Report the sample size, and whether any power or sample size calculations were carried out prior to the main analysis | Section 2.2.1, paragraphs 1-6 | GWAS sources and sample size: Supplementary table 1  Summary level information for observational analyses: Supplementary table 15  Inclusion/exclusion/phenotype definition: Methods | |
|  | c) | Describe measurement, quality control and selection of genetic variants | Section 2.5.4, paragraphs 1  Section 2.6.2, paragraphs 4 | Genetic instruments for MR and MVMR analyses are discussed in the Methods | |
|  | d) | For each exposure, outcome, and other relevant variables, describe methods of assessment and diagnostic criteria for diseases | Section 2.4 paragraphs 1-4  Section 2.2.1, paragraphs 1-6 | GWAS sources and sample size: Supplementary table 1  Summary level information for observational analyses: Supplementary table 15  Inclusion/exclusion/phenotype definition: Methods | |
|  | e) | Provide details of ethics committee approval and participant informed consent, if relevant | Section 2.7.2, paragraph 1  Section 6, paragraphs 1-2 | GWAS used in this study are listed in Supplementary Table 1.  Ethics statements and acknowledgements are in their respective sections in the main text. | |
| 5 | **Assumptions** | Explicitly state the three core IV assumptions for the main analysis (relevance, independence and exclusion restriction) as well assumptions for any additional or sensitivity analysis | Section 1, paragraph 4  Section 2.5 paragraphs 1-2  Supplementary text, Section 1.1 paragraph 1 | Core assumptions are stated Methods and Introduction  Additional MR sensitivity analyses are stated in the supplementary texts | |
| 6 | **Statistical methods: main analysis** | Describe statistical methods and statistics used |  |  | |
|  | a) | Describe how quantitative variables were handled in the analyses (i.e., scale, units, model) | Section 2.5.3, paragraphs 1 | All GWAS are publicly available. Summary of transformation is discussed in the main text. | |
|  | b) | Describe how genetic variants were handled in the analyses and, if applicable, how their weights were selected | N/A | Sources of GWAS are listed in Supplementary Table 1. | |
|  | c) | Describe the MR estimator (e.g. two-stage least squares, Wald ratio) and related statistics. Detail the included covariates and, in case of two-sample MR, whether the same covariate set was used for adjustment in the two samples | Section 2.5.5, paragraphs 3  Section 2.6.2, paragraphs 3 | MR estimates are discussed in the Methods.  Sources of GWAS are listed in Supplementary Table 1. | |
|  | d) | Explain how missing data were addressed | N/A | Only non-missing values are used when conducting observational analysis. | |
|  | e) | If applicable, indicate how multiple testing was addressed | Section 2.6.3, paragraphs 1  Supplementary text, section 1.1, paragraphs 2 | Bonferroni correction was used. This is stated in the Methods and Supplementary text | |
| 7 | **Assessment of assumptions** | Describe any methods or prior knowledge used to assess the assumptions or justify their validity | Section 2.7.1 paragraphs 1-2  Section 4, paragraphs 2 | Observational analyses were performed to supplement the MR results.  Prior knowledge was mentioned in the Discussion.  We also performed MR using alternative markers of kidney function and with different proteomics cohorts. | |
| 8 | **Sensitivity analyses and additional analyses** | Describe any sensitivity analyses or additional analyses performed (e.g. comparison of effect estimates from different approaches, independent replication, bias analytic techniques, validation of instruments, simulations) | Section 2.6.2, paragraphs 3  Section 2.6.5, paragraphs 2  Supplementary text, Section 1.1, paragraphs 1-3, Section 1.2, paragraphs 1, Section 1.3 paragraphs 1-3 | Multiple MR estimates were used and are described in the main text.  We used multiple proteomics cohort.  We performed a series of sensitivity analyses including: steiger test, removing T2D genetic instruments within cis region of target proteins, assess instrument R2, assessing maximum relative bias due to sample overlap, assess colocalization of MR of interest and using eQTL studies to assess potential horizontal pleiotropic effects, and multivariable MR. | |
| 9 | **Software and pre-registration** |  |  |  | |
|  | a) | Name statistical software and package(s), including version and settings used | Section 5, paragraphs 1 |  | |
|  | b) | State whether the study protocol and details were pre-registered (as well as when and where) | N/A | The study protocol was not pre-registered. | |
|  | **RESULTS** |  |  |  | |
| 10 | **Descriptive data** |  |  |  | |
|  | a) | Report the numbers of individuals at each stage of included studies and reasons for exclusion. Consider use of a flow diagram | Section 2.2.1, paragraphs 1-6 | GWAS sample sizes are provided in Supplementary Table 1.  Observational studies reported in Methods | |
|  | b) | Report summary statistics for phenotypic exposure(s), outcome(s), and other relevant variables (e.g. means, SDs, proportions) | N/A | GWAS summary statistics are publicly available.  UK Biobank data are available for approved researchers.  Sources of GWAS are listed in Supplementary Table 1.  MR results are in the Supplementary Table 3, 8. | |
|  | c) | If the data sources include meta-analyses of previous studies, provide the assessments of heterogeneity across these studies | N/A | We did not meta-analyse previous GWAS | |
|  | d) | For two-sample MR:  i.  Provide justification of the similarity of the genetic variant-exposure associations between the exposure and outcome samples  ii.  Provide information on the number of individuals who overlap between the exposure and outcome studies | Section 2.5.1, paragraphs 1  Section 1.3 paragraphs 1  Supplementary text section 1.3 paragraphs 1-2 | Points regarding the similarity of the genetic effects between the exposure and outcome cohort is discussed in the Methods section.  Sample overlap bias discussed in Methods and Supplementary Text | |
| 11 | **Main results** |  |  |  | |
|  | a) | Report the associations between genetic variant and exposure, and between genetic variant and outcome, preferably on an interpretable scale | N/A | Genetic effects of all instruments are reported in Supplementary Table 4 and 5 | |
|  | b) | Report MR estimates of the relationship between exposure and outcome, and the measures of uncertainty from the MR analysis, on an interpretable scale, such as odds ratio or relative risk per SD difference | N/A | MR results are reported in Supplementary Table 3 and 8 | |
|  | c) | If relevant, consider translating estimates of relative risk into absolute risk for a meaningful time period |  | N/A | |
|  | d) | Consider plots to visualize results (e.g. forest plot, scatterplot of associations between genetic variants and outcome versus between genetic variants and exposure) | N/A | MR results are visualized with forest plot/volcano plots. | |
| 12 | **Assessment of assumptions** |  |  |  | |
|  | a) | Report the assessment of the validity of the assumptions | Section 1.1, paragraphs 1-3, Section 1.2 paragraphs 1, Section 1.3 paragraphs 1-2 | This is reported throughout the Results section and Supplementary texts (e.g., heterogeneity results, replication results, coloclalization results) | |
|  | b) | Report any additional statistics (e.g., assessments of heterogeneity across genetic variants, such as *I^2^*, Q statistic or E-value) | Section 3.1.2, paragraphs 3  Section 3.1.5, paragraphs 1 | Heterogeneity (I^2^), and F-statistics are reported in throughout the Results section, and the Supplementary Table 3 and 8 | |
| 13 | **Sensitivity analyses and additional analyses** |  |  |  | |
|  | a) | Report any sensitivity analyses to assess the robustness of the main results to violations of the assumptions | Section 3.1.5 paragraphs 1  Section 3.1.4, paragraphs 1  Section 3.1.3  paragraphs 2  Supplementary text Section 1.1, paragraphs 1-3, Section 1.2 paragraphs 1, Section 1.3 paragraphs 1-2 | Sensitivity analyses and assessment of the different MR assumptions are reported throughout the Results section and supplementary text | |
|  | b) | Report results from other sensitivity analyses or additional analyses | Section 3.1.8, paragraphs 1  Section 3.1.9 paragraphs 1-3  Section 3.1.10, paragraphs 1 | Observational analyses are reported in the Results section | |
|  | c) | Report any assessment of direction of causal relationship (e.g., bidirectional MR) | Section 3.1.1, paragraphs 3  Section 3.1.2, paragraphs 2  Supplementary text, section 1.1, paragraphs 2 | This is in the Results section and Supplementary text | |
|  | d) | When relevant, report and compare with estimates from non-MR analyses | Section 3.1.8, paragraphs 1  Section 3.1.9 paragraphs 1-3  Section 3.1.10, paragraphs 1 | Observational analyses are reported in the Results section | |
|  | e) | Consider additional plots to visualize results (e.g., leave-one-out analyses) | N/A | We used locuszoom plots, Kaplan-Meier curves, and forest plots to visualize results from different sensitivity MR and observational analyses. | |
|  | **DISCUSSION** |  |  |  | |
| 14 | **Key results** | Summarize key results with reference to study objectives |  |  | |
| 15 | **Limitations** | Discuss limitations of the study, taking into account the validity of the IV assumptions, other sources of potential bias, and imprecision. Discuss both direction and magnitude of any potential bias and any efforts to address them | Section 4, paragraphs 8 | Limitation is discussed extensively in Main text | |
| 16 | **Interpretation** |  |  |  | |
|  | a) | Meaning: Give a cautious overall interpretation of results in the context of their limitations and in comparison with other studies | Section 4, paragraphs 8 - 9 | Conclusion provided in Main text. Limitation to the interpretation are discussed in the Methods and Discussion | |
|  | b) | Mechanism: Discuss underlying biological mechanisms that could drive a potential causal relationship between the investigated exposure and the outcome, and whether the gene-environment equivalence assumption is reasonable. Use causal language carefully, clarifying that IV estimates may provide causal effects only under certain assumptions | Section 4, paragraphs 2 | Biological mechanisms described in the main text on the roles of TGF beta, and activin in kidney disease and citations to prior studies showing the relationship between INHBC and kidney disease/diabetic nephropathy based on transcriptome, proteome studies and animal models. | |
|  | c) | Clinical relevance: Discuss whether the results have clinical or public policy relevance, and to what extent they inform effect sizes of possible interventions | Section 4 paragraphs 8 - 9 | The limitation of this study is described in the Discussion section followed by the conclusion drawn from this study | |
| 17 | **Generalizability** | Discuss the generalizability of the study results (a) to other populations, (b) across other exposure periods/timings, and (c) across other levels of exposure | Section paragraphs 2 | Prior evidence based on transcriptome, proteome and animal models are discussed. | |
|  | **OTHER INFORMATION** |  |  |  | |
| 18 | **Funding** | Describe sources of funding and the role of funders in the present study and, if applicable, sources of funding for the databases and original study or studies on which the present study is based | Section 7 paragraphs 1 | Under Funding statement section | |
| 19 | **Data and data sharing** | Provide the data used to perform all analyses or report where and how the data can be accessed, and reference these sources in the article. Provide the statistical code needed to reproduce the results in the article, or report whether the code is publicly accessible and if so, where | Section 5, paragraphs 1 | In Data and code availability section. | |
| 20 | **Conflicts of Interest** | All authors should declare all potential conflicts of interest | Section 8 paragraphs 1 | In Disclosures section | |

This checklist is copyrighted by the Equator Network under the Creative Commons Attribution 3.0 Unported (CC BY 3.0) license.

1.	Skrivankova VW, Richmond RC, Woolf BAR, Yarmolinsky J, Davies NM, Swanson SA, et al. Strengthening the Reporting of Observational Studies in Epidemiology using Mendelian Randomization (STROBE-MR) Statement. JAMA. 2021;under review.

2.	Skrivankova VW, Richmond RC, Woolf BAR, Davies NM, Swanson SA, VanderWeele TJ, et al. Strengthening the Reporting of Observational Studies in Epidemiology using Mendelian Randomisation (STROBE-MR): Explanation and Elaboration. BMJ. 2021;375:n2233.
